# Transcriptomic Characterization of Innate and Acquired Immune Responses in Red-Legged Partridges (*Alectoris rufa*): A Resource for Immunoecology and Robustness Selection

**DOI:** 10.1371/journal.pone.0136776

**Published:** 2015-09-02

**Authors:** Natalia Sevane, Javier Cañon, Ignacio Gil, Susana Dunner

**Affiliations:** Departamento de Producción Animal, Facultad de Veterinaria, Universidad Complutense, Madrid, Spain; Wageningen UR Livestock Research, NETHERLANDS

## Abstract

Present and future challenges for wild partridge populations include the resistance against possible disease transmission after restocking with captive-reared individuals, and the need to cope with the stress prompted by new dynamic and challenging scenarios. Selection of individuals with the best immune ability may be a good strategy to improve general immunity, and hence adaptation to stress. In this study, non-infectious challenges with phytohemagglutinin (PHA) and sheep red blood cells allowed the classification of red-legged partridges (*Alectoris rufa*) according to their overall immune responses (IR). Skin from the area of injection of PHA and spleen, both from animals showing extreme high and low IR, were selected to investigate the transcriptional profiles underlying the different ability to cope with pathogens and external aggressions. RNA-seq yielded 97 million raw reads from eight sequencing libraries and approximately 84% of the processed reads were mapped to the reference chicken genome. Differential expression analysis identified 1488 up- and 107 down-regulated loci in individuals with high IR versus low IR. Partridges displaying higher innate IR show an enhanced activation of host defence gene pathways complemented with a tightly controlled desensitization that facilitates the return to cellular homeostasis. These findings indicate that the immune system ability to respond to aggressions (either diseases or stress produced by environmental changes) involves extensive transcriptional and post-transcriptional regulations, and expand our understanding on the molecular mechanisms of the avian immune system, opening the possibility of improving disease resistance or robustness using genome assisted selection (GAS) approaches for increased IR in partridges by using genes such as *AVN* or *BF2* as markers. This study provides the first transcriptome sequencing data of the Alectoris genus, a resource for molecular ecology that enables integration of genomic tools in further studies.

## Introduction

The red-legged partridge (*Alectoris rufa*) plays a key ecological role as prey of several predator species, many of them considered at risk such as the Imperial eagle (*Aquila adalberti*) or the Bonelli's eagle (*Hieraaetus fasciatus*) [1,2], and is also an important cynegetic species in southern European countries. Despite the widespread distribution of this species (S1 Fig), wild red-legged partridge populations are decreasing [2] and, to face the heavy hunting pressure, millions of captive-reared partridges are released every year. However, restocking with farm-reared partridges may generate new problems concerning conservation and game management of wild populations, to which the possible effects of climate change may be added at a short term [3]. Indeed, the predicted climatic fluctuations may lead to an increase in the distribution and extent of parasitic and infectious processes, as their natural control by low winter temperatures will be reduced. In addition to these problems, restocking with farm partridges has often been criticized for the assumption of an increased risk of disease transmission to wild populations. On the one hand, there is an upward risk of increasing pathogen virulence by successive passes from one animal to another as result of high breeding densities in farms. On the other hand, these infectious agents are often characteristic of animals bred in captivity, and wild individuals have not been exposed to them previously [4–6]. There is also evidence suggesting that medical treatments used in poultry farms to reduce parasite load are ineffective [5,7]. These circumstances, along with the ban of antibiotics for disease prevention and growth promotion by the European Union since 2006 [8], may be risk factors for wild populations after restocking with farm animals.

In comparison with mammals, birds have different repertoires of Toll-like receptors, defensins, cytokines, chemokines, antibodies and other immune molecules. Birds differences also include the presence of heterophils instead of the mammalian neutrophils, lack of lymph nodes, or the presence of the bursa of Fabricius. However, the basic principles of IRs remain constant for all vertebrate species so far studied, including the birds [9, 10]. Many studies have highlighted the large number of genes involved in avian IR, where 400 of the 500 human genes related with the GO term ‘innate immunity’ were identified in chicken [11]. Whole genome approaches have already investigated the avian transcriptomic modifications after experimentally induced immune challenges [12–18], allowing characterization of the full avian transcriptional profile in response to pathogen and external aggressions and opening the possibility of improving disease resistance using genome assisted selection (GAS) approaches.

Thus, directional selection to increase general immunity and hence improving the adaptation of partridges to stress may be a good strategy to obtain ‘robust’ lines which in turn help also to prevent diseases in farms without the use of medical treatments. This study aims to characterize the ability of red-legged partridges to cope with pathogens and other external aggressions after exposure to non-infectious challenges by investigating the transcriptional profiles elicited by extreme high and low immune responses (IR). On the one hand, the innate IR was measured using a skin test with phytohemagglutinin (PHA, a lectin found in plants); PHA stimulates T lymphocytes to release cytokines causing an inflammatory influx of leukocytes and fluid. On the other hand, the acquired IR was quantified using a sheep red blood cells (SRBC) hemagglutination assay, which measures the antibody response integrating the functions of B lymphocytes, helper T lymphocytes, and macrophages. Both methodologies have been widely tested in birds, and are nonlethal and minimally invasive [19]. Selection of those partridges showing extreme IR allowed to investigate the transcriptional profiles underlying the different immune system response using high-throughput mRNA sequencing (RNA-seq). These findings pave the way for improving disease resistance or robustness using GAS approaches in partridges.

## Materials and Methods

### Animals and non-infectious challenges

Starting from 700 partridge chicks (350 males, 350 females) to ensure a minimum number of 300 couples after the breeding period and adequate monogamous pairing, a final set of 600 red-legged partridges were challenged using PHA and SRBC. As no studies using SRBC and PHA challenges have been published for red-legged partridges, we relied on previous chicken experimental designs [20,21] and thus set n = 600 as a reasonable number of samples to detect statistically significant associations and biologically meaningful differences between animals displaying extreme IR. To synchronize and standardize the breeding of the partridges included in the experiment, two time point hatching events separated by one week were selected and the same rearing conditions and diet composition were used for all individuals. Seven month old partridges were subjected to the non-infectious challenges in three consecutive days per week to allow correct assay management according to the available staff.

A detailed explanation on the non-infectious challenges methodology is shown in S1 Data. Briefly, 0.2 ml of a 25% SRBC suspension was injected into the thigh muscle. Blood plasma was collected at day 0 prior to inoculation of SRCB suspension and 6 days after immunization, and was serially diluted in duplicate and incubated with SRBC 0.25% to measure total antibody titers (AbTot). To measure IgG activity, 25 ml of 0.20 M 2-mercaptoethanol in normal saline was added to blood plasma, serially diluted in duplicate and incubated with SRBC 0.25%. The log_2_ of the reciprocal of the greatest dilution of antibody at which there was a visible complex between antigen and antibody was defined as the titer in both cases. A high titer indicates a strong antibody response to the original immunization. At day 5 of the experiment, 0.1 ml of 1.6 mg/ml PHA dissolved in sterile phosphate buffer saline (PBS) was sub-dermically inoculated in the interdigitary skin of one foot, and 0.1 ml of PBS into the other foot. Previously and twenty-four hours (±2 h) after injection, the thickness of the interdigitary skin of each foot was measured and the mitogen stimulation index (MSI) was calculated as the increase in interdigitary skin thickness caused by PHA minus the increase caused by PBS. A large increase in skin thickness indicates a strong T cell-mediated IR.

Twenty-four partridges showing the highest values and 24 showing the lowest values for innate and acquired IR were selected from each hatching group, slaughtering a total of 96 animals at day 7 of the experiment by cervical dislocation. The main avian lymphoid organs–spleen, thymus, bursa of Fabricius, and cecal tonsils- and the skin from the area of injection of PHA were harvested and RNA was preserved using RNAlater (Life Technologies) at -20°C. The non-infectious challenges performed in this study have been widely tested in birds and do not represent any health risk to the animals or the farm [19]. Thus, the 504 non-sacrificed animals were managed according to the farm routine procedures after the non-infectious challenges.

The animal protocol was approved by the Animal Care and Ethics Committee of the Universidad Complutense de Madrid (Spain) (CEA-UCM/32) and birds were handled according to the principles for the care of animals in experimentation established by the Spanish RD 1201/2005 [22].

### RNA extraction and RNA-seq

After non-infectious challenges completion, the 16 females showing the most extreme high and low IR, both innate and acquired, were reselected from the group of 96 and RNA was extracted for RNA-seq analysis. Total RNA was extracted from 25 mg of spleen tissue with the RNeasy Tissue Mini Kit, and from 100 mg of skin from the area of injection of PHA with the RNeasy Fibrous Tissue Kit (QIAGEN, Izasa, Spain) following the manufacturer’s instructions. RNA purity and integrity were assessed by nano-electrophoresis (Bioanalyzer 2100, Agilent Technologies) and optimal RNA Integrity Number (RIN) values were obtained for all samples, ranging between 8.8 and 10.

Four pools were produced consisting each in four equivalent amounts of spleen samples mixed together according to the high (one pool plus replicate) and low (one pool plus replicate) acquired IR groups. The same procedure was also used for producing the four pools corresponding to high and low innate IR groups from skin samples. mRNA libraries were prepared from total RNA samples following Illumina standard protocols (Ilumina, San Diego, USA). Briefly, each total RNA sample (1–4 μg) was treated with DNase, enriched for mRNA using oligo(dT) tags, and fragmented. Samples of poly(A) RNA (0.2–1 μg) were fragmented into 200–500bp and next-generation sequencing (NGS) libraries were prepared, strictly following TruSeq based procedures (Illumina). After size control (mean for all libraries ~280 bp) and quantification, libraries were pooled and bound at a final concentration of about 10 pM to an Illumina SR-flowcell using a Cluster Station apparatus (Illumina). Sequencing was performed in a single-end-read, 1 x 76-base mode on a GAIIx Sequencer (Illumina, Unidad de Genómica, Parque Científico de Madrid) by sequencing-by-synthesis (SBS) chemistry, and running four bar-coded samples per lane (multiplexing). Quality filtering was performed automatically according to Illumina specifications and individual reads were de-multiplexed using the CASAVA pipeline (Illumina v1.8.2), obtaining the FASTQ files used for further bioinformatic analysis. All FASTQ files were submitted to the NCBI Sequence Read Archive (SRA) with the accession number PRJNA268542.

### Bioinformatic analysis of RNA-seq data

For each pool, reads with a quality score of Q > 20 that passed filtering with PRINSEQ (version 0.20.4) [23] were used to generate a complete FASTQ file. The pre-processed reads were mapped to the reference genome sequence of chicken (Galgal4.74) using Bowtie 2 (version 2.2.1) [24] with the parameters—local–D 20 –R 3 –N 1 –L 20 –i S,1,0.50. The SAM file generated by Bowtie 2, which contains the count of reads per base aligned to each location across the length of the genome, was also converted into a binary alignment/map (BAM) format and sorted using SAMTools (version 0.1.18) [25]. The aligned reads were assembled into transcripts and their relative abundance measured using Cufflinks (version 2.1.1) [26] with the parameter—multi-read-correct. The assembled transcripts were merged with the reference annotation (Gallus_gallus.Galgal4.74.gtf, downloaded from Ensembl) using cuffmerge. Differential expression analysis was performed using cuffdiff with the parameter—upper-quartile-norm; the merged assembly and the fragment alignments generated by Bowtie 2 and converted to sorted.bam format with SAMTools were used as input files. Cuffdiff, after estimate how read counts vary for each gene across the replicates, calculates the significance of observed changes in expression: P value (the uncorrected P value of the test statistic), and q value (the false discovery rate (FDR)-adjusted P value of the test statistic). The significance depends on whether P is greater than the FDR after a Benjamini-Hochberg correction for multiple testing (q values between 0 and 0.5 indicate significant changes). The R software application CummeRbund (version 2.6.1) [27,28] was used to visualize the results of the RNA-seq analysis.

### Gene ontology (GO) and KEGG pathway enrichment analyses

GO classification systems were used to assign putative function to each gene by way of biological process, molecular function and cellular components. All analyses were conducted on two independent gene lists containing significantly (0 ≤ q ≤ 0.5) up-regulated and down-regulated genes.

The Database for Annotation, Visualization and Integrated Discovery (DAVID) v6.7b [29] was used to determine pathways and processes of major biological significance through the Functional Annotation Cluster (FAC) tool based on the GO annotation function. High stringency ease score parameters were selected to obtain confident enrichment scores.

The algorithm for the reconstruction of accurate cellular networks (ARACNE) method was also used to identify co-regulated gene pairs of high statistical significance by using mutual information and data processing inequality (DPI) to eliminate indirect relationships [30]. After constructing gene regulatory networks with ARACNE, the Cytoscape´s (version 3.1.1) [31] plugin BiNGO (v.2.44) [32] was used for GO pathway enrichment analyses. Hypergeometric test was used to identify overrepresented GO pathway terms with a significance level at 0.05 and Benjamini-Hochberg method was used for the correction of the p-values.

Kyoto Encyclopedia of Genes and Genomes (KEGG) pathway tool was used through DAVID online tools to visually map clusters of the partridge genes involved in common pathways and processes for both pathway-specific and molecular overview purposes.

### Real-time PCR validation

To confirm RNA-seq data, 5 of the regulated genes in the spleen tissue (*AVD*, *CTSD*, *NOV*, *SOX13*, and *SPTSSA*) and 9 in the area of injection of PHA (*ADAMTSL1*, *ATP12A*, *AVD*, *CD3E*, *CD7*, *CTSD*, *GSAP*, *MAD2L1*, and *UBASH3A*) were selected for further validation by qRT-PCR. A total of 10 samples per tissue, 5 biological replicates corresponding to the individuals displaying the highest IR and 5 corresponding to the lowest IR (all included in the RNA-seq pools) were used. The total RNA was used for RT-cDNA synthesis using Superscript II First Strand cDNA Synthesis kit (Invitrogen, Spain). With the resulting cDNA template, real-time assays were conducted for all target genes and three commonly reference genes–*ACTB*, *GAPDH*, *G6PDH-*, using an Eco Real-Time PCR System (Illumina, Cultek, Spain). A master mix was prepared using the Dynamo HS SYBR Green qPCR Kit (Finnzymes, Fisher Scientific, Spain). The expression level and stability of the reference genes were measured using NormFinder [33]. The PCR primers were designed using primer 3 (http://bioinfo.ut.ee/primer3-0.4.0/primer3/) (S1 Table). Real-time PCR reactions were performed in 5 μl reaction volume, with 10 mM of each primer and 0.5 μl of 10-fold cDNA dilutions. Reactions for standard curves, sample assays and no-template controls were carried out in triplicates. The quantification program consisted of 40 cycles of 95°C for 10 s and 30 s at annealing temperature, ending with a melt curve of 15 s at 95°C, 15 s at 55°C and 15 s at 95°C, and continuous fluorescence measurement. The results were exported into EcoStudy Software v4.0 (Illumina) to calculate and normalize the expression of each gene.

The target gene data were analysed using the Relative Expression Software (REST), which follows the Pfaffl method [34], normalizing their expression levels through the three reference genes (*ACTB*, *GAPDH*, *G6PDH*). When differences between samples of either IR magnitude (low or high) were subjected to random, a test was carried out and the alternative hypothesis was accepted for a P value lower than 0.09.

### Statistical analysis

Gender differences were determined by variance analysis, using the GLM procedure, considering sex as a unique effect, and the Scheffe’s multiple-comparison procedure at α = 0.005. The correlations between MSI, AbTot and IgG, and between the data of RNA-seq and qPCR were analysed by the Spearman’s test using the CORR procedure of SAS. All the statistical analyses were carried out using the SAS statistical package v. 9.1.3 [35].

## Results

### Non-infectious challenges

There were significant differences between the magnitude of the acquired IR of males and females, both for AbTot and IgG titers, with the females showing stronger antibody responses after immunization with SRBC (Table 1). As expected, AbTot titers correlated positively with IgG titers (r = 0.86, P < 0.001). The innate IR measured by MSI after inoculation of PHA in the interdigitary skin showed no sex differences (Table 1). There was no significant correlation between innate MSI and acquired antibody titer values.

**Table 1:** Gender differences in total antibody (AbTot) and IgG titers, and mitogen stimulation index (MSI) after non-infectious challenges with sheep red blood cells (SRBC) and phytohemagglutinin (PHA), respectively. Values are expressed as means ± standard deviation: titer as log_2_ of the reciprocal of the greatest dilution causing visible hemagglutination; skin thickness in cm.

Variable	Females	Males
Total antibody titer (AbTot)	7.34 ± 1.93^a^	6.57 ± 1.84^b^
IgG titer	5.75 ± 1.94^a^	5.04 ± 1.89^b^
Mitogen stimulation index (MSI)	0.26 ± 0.22^a^	0.23 ± 0.21^a^

^a-b^ Within row, values with the same letter are not significantly different (P>0.005).

### Summary of the RNA-seq data set

Given the significantly higher acquired IR displayed by females, only partridge female samples were included in downstream analysis. RNA-seq analysis was performed to identify genes that might be involved in the differential transcriptional responses between animals with high and low IR and obtain a broad view on the associated cellular processes. Two biological replicates of both high and low IR spleen and skin samples, consisting of RNA pools resulting from mixing equal amounts of four different RNA samples, were used. High-throughput sequencing generated 8.0–13.7 million (M) raw reads for each sample and more than 99% of them passed quality filtering (Q > 20) (Table 2). Filtered reads were mapped to the chicken genome using Bowtie 2 software. A total of 96.1 M reads were processed with Bowtie 2 and the percent of mapped reads was similar among different samples (79.1–86.2%). The number of unique mapping events generated by Bowtie 2 was 59.5 M, representing 61.7% of the total alignment.

**Table 2:** Statistics for the filtering and mapping of reads to the reference chicken genome.

Sample^1^	SPL1	SPL2	SPH1	SPH2	SKL1	SKL2	SKH1	SKH2
Total reads (M)	13.0	13.7	13.4	12.2	13.2	11.0	12.4	8.0
Good reads (M)	12.9	13.6	13.3	12.1	13.1	10.9	12.3	7.9
% Good reads	99.2	99.4	99.3	99.2	99.2	99.1	99.2	99.4
Mapped reads (M)	11.2	11.5	11.4	10.0	11.0	8.6	10.4	6.6
% Overall alignment rate	86.2	84.77	85.5	82.9	83.7	79.1	84.1	83.4
Unique mapping (M)	8.2	8.6	8.4	7.5	7.9	6.4	7.6	4.9
% Unique mapping	63.6	63.4	63.0	61.6	60.2	58.2	61.9	61.6
Multiple mapping (M)	2.9	2.9	3.0	2.6	3.8	2.3	2.8	1.7
% Multiple mapping	22.7	21.4	22.5	21.3	23.5	20.9	22.9	21.8

^1^Complete sample names: SPL1, spleen low IR 1; SPL2, spleen low IR 2; SPH1, spleen high IR 1; SPH2, spleen high IR 2; SKL1, skin low IR 1; SKL2, skin low IR 2; SKH1, skin high IR 1; SKH2, skin high IR 2.

The mapping data generated by Bowtie 2 was processed with Cufflinks toolkits for transcript assembly and differential expression analysis. The detected transcripts were 52,344 in spleen and 47,669 in skin, and among them, 9,565 and 9,743 different annotated transcripts were identified, respectively, with 8,732 common transcripts between both tissues (Table 3).

**Table 3:** Statistics of genes differentially expressed between individuals with high and low IR.

Classification	SPL-SPH^1^	SKL-SKH^1^	Common Spleen-Skin genes
Detected genes (XLOC id’s)	52,344	47,669	34,612
Annotated genes	9,565	9,743	8,732
Detected isoforms (TCONS)	78,903	71,736	50,547
Up-regulated genes	78	1,410	7
Down-regulated genes	19	88	0
Differentially expressed isoforms	83	1,421	3
Differentially spliced genes	14	11	1
Genes with promoter-switching	7	17	0

^1^SPL, spleen low IR; SPH, spleen high IR; SKL, skin low IR; SKH, skin high IR.

**Fig 1:**
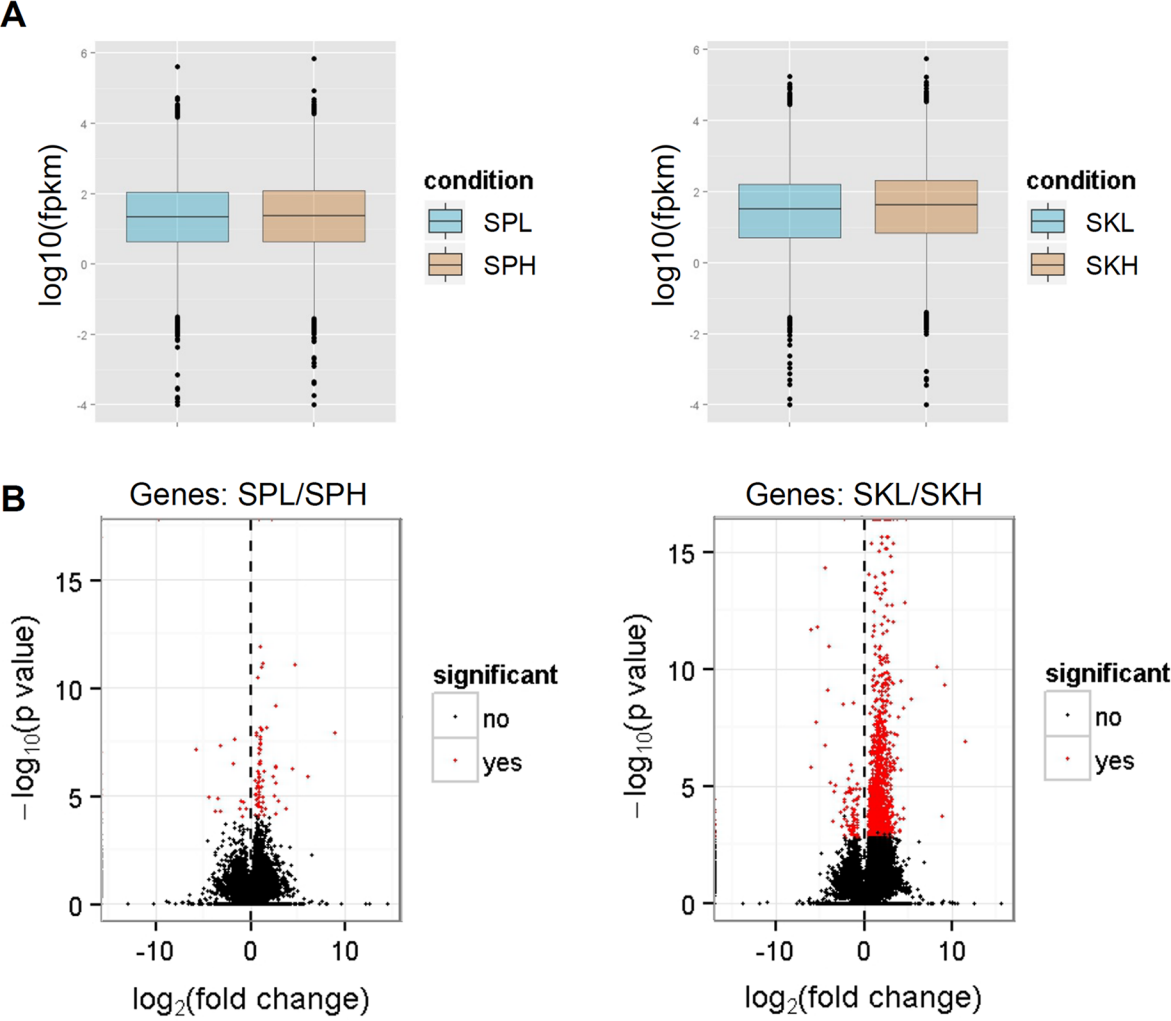
Summary of RNA-seq data from spleen and skin samples from animals displaying extreme IR. (A) Box plot showing the distribution of fragments per kilobase of exon per million fragments mapped (FPKM) values. (B) Volcano graph showing differentially (in red) and non-differentially (in black) expressed genes. Values of >0 correspond to down-regulated genes, while values of <0 correspond to up-regulated genes. SPL, spleen low IR; SPH, spleen high IR; SKL, skin low IR; SKH, skin high IR.

**Fig 2:**
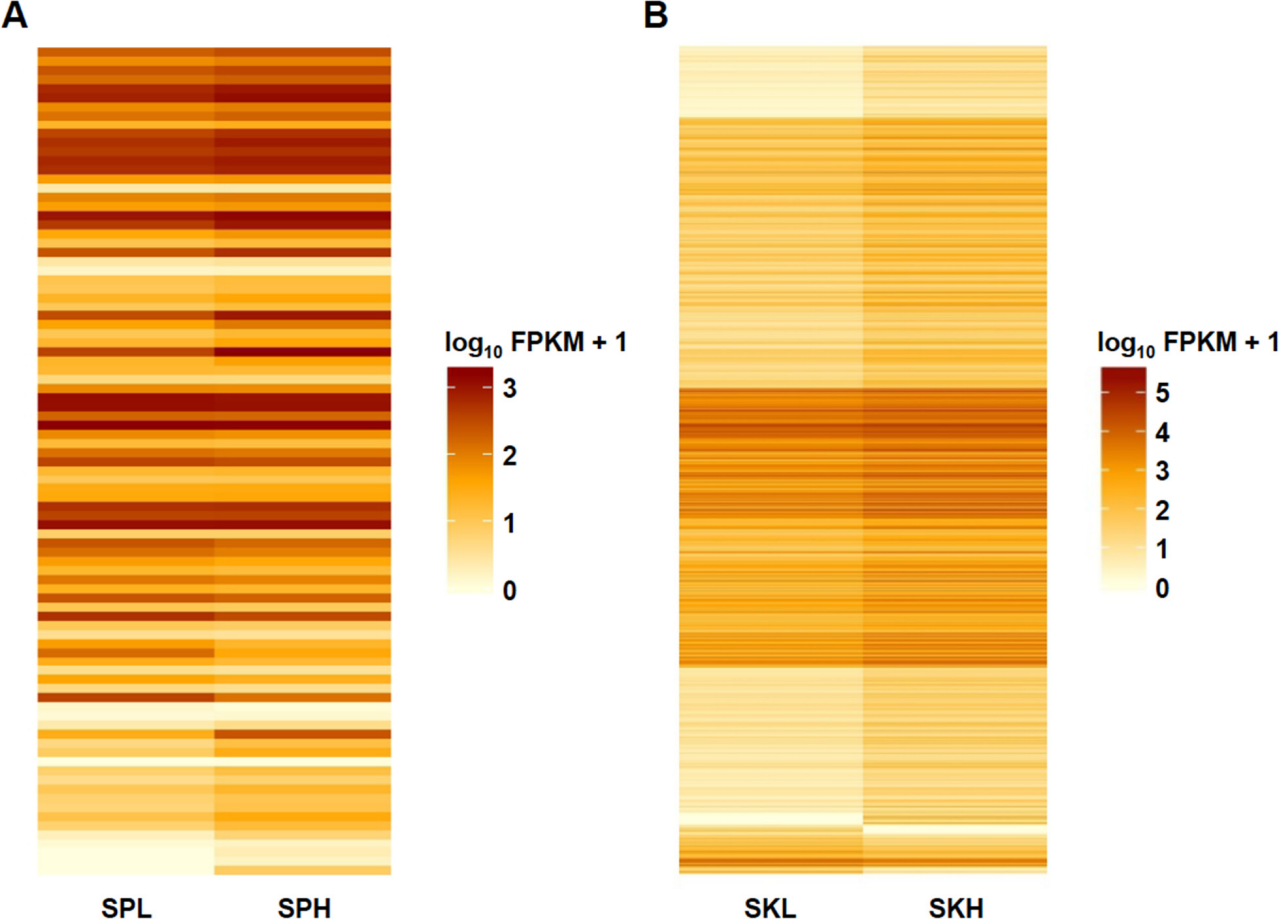
Differential gene expression heatmap from RNA-Seq data. SPL, spleen low IR; SPH, spleen high IR; SKL, skin low IR; SKH, skin high IR.

The abundance of gene transcripts was expressed as the number of reads mapping to a gene divided by the gene length in kilobases and the total number of mapped reads in millions-fragments per kilobase of exon per million fragments mapped (FPKM)- [36] (Fig 1A). The ‘volcano plot’ in Fig 1B relates the observed differences in gene expression to the significance associated with those changes under Cuffdiff's statistical model. Gene expression differences among extreme IR were higher in skin samples, involving 1,410 up-regulated genes (higher transcript levels in high IR samples than in low IR samples) and 88 down-regulated (Fig 2B, Table 3, S2 Table). In the case of spleen samples, up-regulated genes summed up 78 and down-regulated 19 (Fig 2A, Table 3, S2 Table). Regarding differentially spliced genes and genes with promoter-switching, the results were similar in both tissues and ranged from 7 to 17 (Table 3).

### Functional analysis of immune response

**Fig 3:**
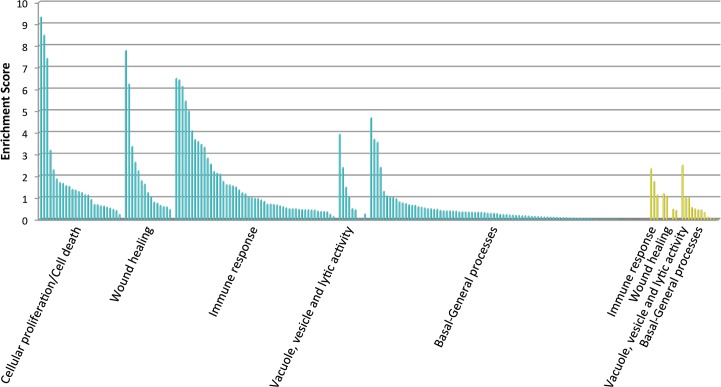
Functional analysis of differentially expressed genes between partridges with high and low IR. DAVID Functional Annotation Cluster (FAC) analysis was conducted on two independent gene lists containing 509 up-regulated genes and 40 down-regulated genes (0 ≤ q ≤ 0.5) under high stringency ease scores. *In black*: grouped FACs for up-regulated genes. *In grey*: grouped FACs for down-regulated genes.

To obtain a comprehensive picture of the pathways and biological processes switched on/off as the IR grows stronger, the expression data was analysed using the DAVID tool. Among the 1,410 and 78 up-regulated sequences in skin and spleen samples respectively, 585 shared significant homology with chicken genes encoding proteins of known function (S3 Table). However, 3 of them matched two or more genes and were not considered for downstream analysis. Finally, DAVID FAC analysis included 509 up-regulated sequences in 187 clusters in the up-regulated group mainly for cellular proliferation, cell death, wound healing, immune response, and lytic activity processes (Fig 3, S4 Table). Among the 88 and 19 sequences down-regulated in skin and spleen samples, respectively, 47 shared significant homology with different chicken genes encoding proteins of known function (S3 Table). DAVID FAC analysis included 40 down-regulated sequences in the analysis and retrieved 19 clusters with lower enrichment scores (Fig 3, S4 Table).

Gene regulatory networks were constructed with ARACNE method and analysed with Cytoscape (S2 Fig). As with DAVID FAC analysis, the most represented up-regulated processes are involved in regulation of the immune response and cellular proliferation, including ‘immune system process’, ‘regulation of lymphocyte activation’, ‘regulation of T cell activation’, ‘hemopoiesis’, or ‘cell cycle process’ (S3A Fig); whereas the main down-regulated processes are related to basal or general functional categories, such as ‘organic acid catabolic processes’, ‘carboxylic acid catabolic process’ or ‘arginine metabolic process’ (S3B Fig).

The KEGG pathway enrichment analysis retrieved a total of 23 pathways for the up-regulated group of genes (S5 Table). ‘Focal adhesion’ was the most significantly enriched pathway, with 25 of the associated genes up-regulated by higher IRs. The map showing the genes associated with (A) ‘Focal adhesion’, (B) ‘Cytokine-cytokine receptor interaction’, (C) ‘T cell receptor signalling pathway’, (D) ‘Cell cycle’, (E) ‘Lysosome’, (F) ‘ECM-receptor interaction’, (G) ‘Natural killer cell mediated cytotoxicity’, (H) ‘Leukocyte transendothelial migration’, (I) ‘Jak-STAT signalling pathway’, (J) ‘Hematopoietic cell lineage’, (K) ‘B cell receptor signaling pathway’, (L) ‘Fc epsilon RI signalling pathway’, (M) ‘Fc gamma R-mediated phagocytosis’, and (N) ‘VEGF signalling pathway’ are shown in S3 Fig.

Finally, we also analysed the distribution of significantly regulated genes along the chicken chromosomes for the group of annotated partridge sequences, confirming four enriched genomic regions in chromosomes 1, 28, 26, and 9 (S6 Table). Among the functional clusters retrieved by the FAC DAVID analysis on the groups of genes from these enriched genomic regions, it is worth highlighting the clusters related to ‘cytokine receptor activity’, ‘regulation of leukocyte and T cell activation’, ‘cytoskeleton organization’, ‘hemopoiesis’, ‘immunoglobulin’, and ‘focal adhesion’ in chromosome 1 (S6 Table, sheet Chr1).

### Differential splicing and alternative promoter usage

Alternative splicing of 24 different loci -11 genes in skin and 14 in spleen samples-, along with the same miRNA (gga-mir-6599) in both tissues, was found between high and low IR (Table 3, S7 Table). Results of functional clustering reveal that these differentially spliced genes are classified into six functional clusters in which ‘regulation of transcription’ is the most highly represented biological process (S7 Table).

Differences in promoter switching of other 24 loci -17 genes in skin and 7 in spleen samples- were found between extreme IRs (Table 3, S8 Table). DAVID FAC analysis classified these loci into five functional clusters in which ‘alternative splicing’ was the most significantly enriched process (S8 Table).

### Validation of RNA-seq data by real-time RT-PCR

In order to validate the expression profiles from RNA-seq analysis, qRT-PCR was performed to determine the expression levels of 5 regulated genes in spleen tissue-*AVD*, *CTSD*, *NOV*, *SOX13*, *SPTSSA*-, and 9 in the area of injection of PHA-*ADAMTSL1*, *ATP12A*, *AVD*, *CD3E*, *CD7*, *CTSD*, *GSAP*, *MAD2L1*, and *UBASH3A*-. To avoid bias, the tested genes were chosen from an array of different processes including immune system processes (*ADAMTSL1*, *AVD*, *CD3E*, *CD7*, *UBASH3A*), lytic activity (*CTSD*), wound healing (NOV), cellular proliferation (*MAD2L1*), and basal or general biological processes (*GSAP*, *SPTSSA*, *ATP12A*). Also, to obtain reliable qRT-PCR results, expression normalization with three commonly used housekeeping genes was performed (*ACTB*, *GAPDH*, and *G6PDH*).

The qRT-PCR expression results were significant for all tested genes, except for *CTSD* gene in spleen samples (S9 Table), and highly correlated with the expression profiles from RNA-seq analysis (r = 0.85, P < 0.0001) (Table 4).

**Table 4:** Comparison between RNA-seq data and qRT-PCR results.

Tissue	Gene Symbol	Gene Name	Fold Change	
			qPCR	RNA-seq
Spleen	*AVD*	Avidin	1.41	0.62
	*CTSD*	Cathepsin D	0.64	0.52
	*NOV*	Nephroblastoma overexpressed gene	1.37	0.67
	*SOX13*	SRY (sex determining region Y)-box 13	-1.88	-4.48
	*SPTSSA*	Serine palmitoyltransferase small subunit A	-2.00	-3.42
Skin	*ADAMTSL1*	ADAMTS-like 1	-2.49	-1.18
	*ATP12A*	ATPase H+/K+ transporting nongastric alpha polypeptide	-8.00	-1.35
	*AVD*	Avidin	1.62	1.17
	*CD3E*	CD3e molecule epsilon (CD3-TCR complex)	3.86	2.21
	*CD7*	CD7 molecule	3.81	2.68
	*CTSD*	Cathepsin D	0.53	1.02
	*GSAP*	Gamma-secretase activating protein	1.57	4.51
	*MAD2L1*	MAD2 mitotic arrest deficient-like 1 (yeast)	1.95	2.07
	*UBASH3A*	Ubiquitin associated and SH3 domain containing. A	4.79	2.59

## Discussion

Given the reduction of wild red-legged partridge populations and the millions of captive-reared partridges released every year in Southern European countries, present and future challenges for wild populations include the resistance against possible disease transmission after restocking, and the need for adaptation to the stress prompted by the introduction into a new habitat. Disease resistance has been postulated to be a multigenic trait, regulated by the immune system and influenced by interactions with physiological and environmental factors [37]. The integration of neuroendocrine and immune systems is well established and is reflected in covariation between stress and immune associated diseases [38–41]. Also, the genetic background of an individual has been shown as an important factor in the orchestration of IR [20], which opens the possibility of improving disease resistance using GAS approaches.

The innate IR has a crucial and controlling role in the ability to resist infection. It provides an important initial response to pathogens, and also determines the course of adaptive IR and hence of immunological memory. However, selection for an improved adaptive IR against specific diseases may compromise the ability to mount an appropriate response against a different pathogen [9,42]. Thereof, a strategy based on selection for increased innate IR may improve general immune robustness, reinforcing the ability to resist infection by a wide spectrum of pathogens.

Primary immune organs such as the bursa of Fabricius (the site of B lymphocyte maturation in birds) and thymus (the site of T lymphocyte maturation) provide important data regarding immunological development. However, and as the experimental animals were seven months old, these organs had started involutioning at the time of the experiment and were not available for all individuals. Thus, we opted for analysing spleen tissue with RNA-seq. This secondary lymphoid organ combines the innate and adaptive immune system in a unique way [43]. However, the differential gene expression profile obtained from spleen samples was poor compared with that of skin tissue, probably due to the bias that the differences on the status of involution of the bursa of Fabricius and thymus among individuals was producing. Given the implication of spleen in both innate and adaptive IR, we included the scarce differentially expressed and annotated genes from this tissue (S3 Table) along with the results obtained for skin in the GO analysis. Regarding the sex differences in immune function detected here, with partridge females exhibiting higher acquired IR than males, they have been already well established in vertebrates [44].

We demonstrated that non-infectious challenges with SRBC and PHA allow the classification of red-legged partridges according to the magnitude of their IR and the characterization of the transcriptional profiles implicated in these differences. This is the first study integrating both non-infectious challenges and RNA-seq analysis in partridges, offering a wide immunogenetic picture as a resource for molecular ecology of a wild bird species and further investigation of immune-specific signalling networks in birds in general. A total of 1,410 up- and 88 down-regulated genes in skin, and 78 up- and 19 down-regulated genes in spleen were identified. These differentially expressed genes are involved in many crucial pathways and biological processes implicated in the orchestration of IR, and can be potentially used as molecular markers for characterization of IR in different avian species. However, a large proportion (~60%) of differentially expressed sequences lacked a functional annotation, in concordance with similar studies [16,45]. The unmapped component is a potential source of important information that may represent novel IR genes in birds, thus further analyses need to be done in subsequent studies to uncover their function.

The results obtained in this study supported the down-regulation of processes related to basal or general functional categories as the IR grows stronger, e.g. ‘organic acid catabolic processes’, or ‘carboxylic acid catabolic process’ (S3B Fig). On the contrary, up-regulated genes fell in four main classes (Figs 3 and S3): a) cellular proliferation and cell death; b) wound healing; c) immune response processes; and d) lytic activity. The different categories of up-regulated genes are discussed below.

### Cellular proliferation and cell death

Cellular proliferation processes showed the highest enrichment scores in the FAC analysis of up-regulated genes (Fig 3). DAVID identified 145 genes with fold changes of gene expression ranging from 0.46 to 4.63 that functionally clustered into common GO terms related to this category, such as cell cycle phases, mitotic cell cycle, organelle fission, kinetochore, DNA packaging, etc., but also regulation of programmed cell death (S4 Table).

The important overexpression of cellular proliferation processes is expected given their key role in the progression of the IR against an external aggression or pathogen. In fact, 40% of the genes included in pathways related to cellular proliferation play also roles in different IR processes, some of them directly implicated in leukocyte proliferation, as for example: i) *CD3E*, essential for early thymocyte maturation, differentiation and proliferation of T lymphocytes [46]; ii) the T lymphocyte membrane protein CD5 (*CD5*), which plays a key role in B1 cell physiology [47]; iii) the protein kinases *PRKCQ* and *SYK*, with important roles from controlling cell growth and proliferation to the initiation and regulation of immunological responses [48]; or iv) *TNFSF4*, also known as *OX40L*, which promotes T cells proliferation, survival and differentiation, and regulates their cytokine production [49] (S4 Table).

On the other hand, the immune system is highly dependent on cell death for efficient responsiveness of many immune cell types during innate, humoral and cellular IRs, as well as for proper adaptive immune self-tolerance and homeostasis [50]. Consequently, overexpression of genes implicated in the regulation of cell death, such as the nuclear apoptosis-inducing factor 1 (*NAIF1*) or the death-associated protein kinase 1 (*DAPK1*), was also detected in the partridges displaying higher IR.

### Wound healing

Skin wound healing process comprises three partially overlapping phases [51]: i) blood clotting and inflammation; ii) formation of new tissue through reepithelialisation and development of granulation tissue; and finally iii) tissue remodelling. DAVID analysis identified 68 up-regulated genes with fold expression changes ranging from 0.43 to 3.75 that functionally clustered into common GO terms related to this category, such as extracellular matrix organization, collagen formation, skin development, and tissue regeneration (Fig 3, S4 Table). However, GO enrichment scores did not reveal any pathway overrepresented for blood clotting, probably because blood vessels were not directly injured in this assay.

Immune cells modulate wound healing and scar formation by promoting cellular cross-talk, secreting signalling molecules such as cytokines and chemokines, and growth factors (see Immune response below) [52]. Thus, as happens for cellular proliferation, many genes (63%) included in pathways related to wound healing play also roles in different IR processes (S4 Table).

An example of a transcription factor up-regulated upon injury in this study and implicated in the reepithelialisation phase is the c-Fos induced growth factor (*FIGF*) [53]. Another example is the E2F family of transcription factors, important regulators of cell proliferation through their effects on the expression of genes involved in cell cycle progression [54]: *E2F1*, up-regulated in this study, has been implicated in correct reepithelialization and inflammatory response [55]; *E2F5*, also overexpressed in the ‘robust’ group of partridges (high IR), seems to play important roles during keratinocyte maturation [56]. One of the major regulators of wound repair is transforming growth factor b (*TGF-β*), which affects different cell types in the healing wound [57] in part through its receptors *TGFBR2* and *TGFBR3*, both of them up-regulated in the group of partridges showing higher IRs.

Finally, the resulting dermal tissue after the remodelling phase of wound healing is mainly composed of extracellular matrix, which undergoes significant remodelling, as can be seen in the high enrichment score of the GO term ‘extracellular matrix’ (S4 Table). It includes genes related to collagen production and organization (*COL6A3*, *COL6A2*, *COL6A1*, etc.), or matrix metalloproteinases (MMP) involved in extracellular matrix remodelling (*MMP7*, *MMP23B*), and previously implicated in infection response [45].

### Immune response

DAVID analyses identified 198 up-regulated genes with fold changes of gene expression ranging from 0.34 to 4.63 that functionally clustered into common GO terms related to immune system processes, including regulation of leukocyte proliferation, differentiation and activation, hemopoietic or lymphoid organ development, thymic T cell selection, or IR-activating signal transduction (S4 Table).

Belonging to the first line of defence of the avian innate immunity and displaying antibacterial activity, the lysozyme (*LYZ)* and the transferrin receptor (*TFRC)* genes [58] were both overexpressed in the high IR group of samples. Collectin (*COLEC12)*, which acts as opsonin and enhances phagocytosis, was also up-regulated, as well as the avian-specific avidin (*AVN*), over-expressed both in spleen and skin samples, and whose induction has been shown to occur in response to infection, tissue injury, or inflammation [16,59].

The overexpressed cytokine-related genes in partridges with higher IR included: i) several interferon (IFN)-regulated genes (IRGs) (*IFNAR1*, *IFNAR2*, *IRF2BP2*, *IFI30*, *IRF*-4, *STAT4*, *JAK3*, *JAKMIP1*, *SOCS5*, *TRIM59)*; ii) interleukin (ILs) receptors (*IL2RA*, *IL9R*, *IL21R*, *IL7R*, *IL2RG*) with functional activity involving lymphocytes; iii) the colony-stimulating factor (CSFs) receptor *CSF1R*; iv) transforming growth factor (TGFs) receptors (*TGFBR2*, *TGFBR3*) with crucial roles in the regulation of inflammatory responses; v) tumor necrosis factors (TNFs) superfamily members (*TNFRSF13C*, *TNFAIP8L1*, *C1QTNFG*, *TNFSF4*, *TNFRSF18*, *TNFRSF1B*, *TNFRSF9*, *TNFRSF25*), which display a wide spectrum of activities including both the regulation of IRs and the development of the architecture of the immune system and other organs; and vi) small peptide chemokines (*CCR4*, *CCL19*, *CCR8*, *CXCR5*, *CXCL14*).

The IFN-mediated innate IR provides a robust first line of defence against invading pathogens. Following pathogen detection and subsequent IFN production, IFN molecules bind to cell surface receptors and initiate a signalling cascade through the Janus kinase signal transducer and activator of transcription (JAK-STAT) pathway, up-regulated in the present study (S3-I Fig), leading to the transcriptional regulation of hundreds of IRGs [60]. IFN signalling also plays an important role in shaping the adaptive IR. Increased expression of IFN-α receptor 1 (*IFNAR1*) and 2 (*IFNAR2)* subunits was detected in partridges showing high IR. Other overexpressed genes were *JAK3*, a member of JAKs that plays an important role in the differentiation of hematopoietic cells [61], and *STAT4*, which belongs to a family of transcriptional activators that drive IRG expression [60].

Pattern-recognition receptors (PRRs) reinforce IFN signalling and prime cells for enhanced pathogen detection. From the variety of PRRs expressed on avian immune cells, increased expression of the type 1 isoform of *TLR2* (*TLR2-1*), *TLR6*, and the avian-specific *TLR21* Toll-like receptors (TLRs) was detected. The TLR family act as a mechanism of connection between the innate and adaptive immune systems [62].

Among chemokines, the over-expressed pattern of the chemokine (C-X-C motif) ligand 14 (*CXCL14)* detected here is particularly interesting because of its known roles in inflammatory responses. This chemokine mediates its antimicrobial role via selective activity in monocytes, dendritic cells, and natural killer cells [63], and provides an instant antimicrobial system that safeguards the skin from excessive cellular IRs [64].

Up-regulation of *CD4* gene was also detected. This gene encodes a membrane glycoprotein of T lymphocytes, B cells, macrophages, and granulocytes that interacts with major histocompatibility complex (MHC) class II antigens. Several genes related to MHC class I (*BFIV21*, also known as *BF2*) and class II (*BLB1*, also known as *B-LB*, and *DMB2*, also known as *HLA-DMB*) were also up-regulated. The MHC is implicated in antigen processing and presentation to T lymphocytes and natural killer cells. Compared with placental mammals, the avian MHC shows a single classical class I (BF2, major histocompatibility complex class I antigen BF2) and class II (B-LB, major histocompatibility complex class II beta chain) molecules highly expressed, despite having multiple MHC genes [65–67]. The dominantly expressed class I BF2 molecule can determine decisive resistance and susceptibility to several infectious pathogens, leading to strong genetic associations with infectious disease [66], which is in agreement with its detected overexpression in partridges with a greater ability to mount stronger IRs. *DM* genes are located in the MHC B region of avian genomes and include the *DMB2* gene (major histocompatibility complex, class II, DM beta), which encodes one class II DM β chain [68] and is overexpressed in partridges showing higher IRs. In mammals, the DM molecules execute key functions in the class II antigen presentation pathway.

Immunoglobulins recognize foreign antigens and initiate IRs such as phagocytosis and the complement system. Several immunoglobulin genes were up-regulated in the robust group of partridges, including *TIMD4*, *CD79B*, *IGJ*, *IGSF10*, *VSIG4*, and *IGSF6*.

Overexpression of several members of the DOCK family (*DOCK2*, *DOCK8*, *DOCK10*, and *DOCK11*) implicated in the regulation of IR was also detected.

Besides all this positive regulation of immune related processes, negative regulators of signalling are also required to resolve the IFN-induced state and facilitate the return to cellular homeostasis. Thus, the down-regulation of some immune related genes (S4 Table) and the overexpression of suppressor elements help to maintain a controlled cytotoxic IR. For example, *JAKMIP1*, implicated in the containment of T cell-mediated cytotoxicity [69], was overexpressed, as well as *SOCS5*, a suppressor of cytokine signalling induced early in the IFN response [60], or *TRIM59*, which negatively regulates upstream kinases for IRGs [70].

### Lytic activity

Finally, DAVID analyses identified 82 up-regulated genes with fold changes of gene expression ranging from 0.52 to 3.74 that functionally clustered into common GO terms related to peptidase activity, lysosome, lytic vacuole, or cytoplasmic vesicle processes (S4 Table). Forty percent of genes displayed both lytic and immune roles, explained by the lytic activity that immune cells such as macrophages, heterophils and natural killer cells develop during innate IR. S3-E Fig shows the overrepresented ‘Lysosome’ KEGG pathway that includes up-regulated genes such as cathepsin C (*CTSC*), D (*CTSD*), and S (*CTSS*) with important lytic activities, or clathrin light chain (Lca) (*CLTA*), that plays a major role in the formation of coated vesicles.

## Conclusions

A comprehensive immunogenic picture of partridge transcriptional profiles elicited by non-infectious challenges with PHA and SRBC has been obtained in this study. The analysis of the pathways and biological processes switched on/off as the IR grows stronger has uncovered the pivotal role of the immune system around which cellular proliferation, cell death, wound healing and lytic activity processes are orchestrated in the different phases of the IR. The immune-regulated genes uncovered are key components of crucial biological processes and pathways, such as ‘immune system process’, ‘regulation of lymphocyte activation’, ‘regulation of T cell activation’, ‘hemopoiesis’, or ‘cell cycle process’. Our results indicate also that partridges displaying higher IR have enhanced activation of host defence gene pathways complemented with a tightly controlled desensitization that facilitates the return to cellular homeostasis. These data open the possibility of improving disease resistance or robustness using GAS approaches for increased IR in partridges by using genes such as *AVN* or *BF2* as markers, reinforcing the ability to resist infection by a wide spectrum of pathogens and to cope with the stress prompted by new dynamic and challenging scenarios. This work also provides the first transcriptome sequencing data of the Alectoris genus, a resource for molecular ecology that enables integration of genomic tools in further studies.

## Supporting Information

S1 Data Non-infectious challenges methodology and timetable. (DOCX)

S1 Fig Red-legged partridge (*Alectoris rufa*) geographical distribution (The IUCN Red List of Threatened Species, 2014, www.iucnredlist.org). (PDF)

S2 Fig Gene regulatory networks and gene ontology (GO) enrichment analysis for genes significantly (A) up-regulated and (B) down-regulated between animals with high and low IR. The size of circles is proportional to the number of genes associated with the GO term. The arrows represent the relationship between parent–child terms. The colour scale indicates corrected p-value of enrichment analysis: uncoloured nodes are not overrepresented, but they are the parents of overrepresented categories further down; yellow nodes represent GO categories that are overrepresented at the significance level; for more significant p-values, the node colour gets increasingly more orange. Both A1 and A2 figures are connected by an arrow (*) from ‘biological regulation’ to ‘regulation of biological processes’.
(PDF)

S3 Fig KEGG pathway maps of up-regulated partridge genes involved in common pathways and processes in animals displaying high IR. Red stars indicate the genes differentially expressed included in the pathway.
(PDF)

S1 Table Housekeeping and target genes used in the real-time PCR assay indicating locus symbol, GenBank accession number, and primer sequence. (DOCX)

S2 Table Loci differentially expressed between animals with extreme IR in spleen (sheet SPLEEN) and skin (sheet SKIN). (XLSX)

S3 Table Gene list report on the 582 up-regulated and 47 down-regulated (0 ≤ q ≤ 0.5) partridge sequences that shared significant homology with chicken genes encoding proteins of known function. *In blue*: differentially expressed genes in spleen. *In orange*: differentially expressed genes in skin. *In dark blue or orange*: genes for which DAVID Functional Annotation Cluster (FAC) analysis produced enriched functional clusters under high stringency conditions (see S4 Table).
(XLSX)

S4 Table Results for the 509 up-regulated (UP sheet) and 40 down-regulated (DOWN sheet) genes for which annotation information was available at DAVID (http://david.abcc.ncifcrf.gov/) in September 2014, including the corresponding DAVID scores (P values) and the lists of genes in every significant category. (XLSX)

S5 Table Enriched KEGG pathways for up-regulated partridge genes in animals displaying high IR. (XLSX)

S6 Table Distribution of enriched genomic regions along the chicken chromosomes and enriched functional clusters under high stringency conditions for chromosome 1 (sheet Chr1), 9 (Chr9), 28 (Chr28), and 26 (Chr26) obtained with DAVID. (XLSX)

S7 Table Genes exhibiting differential splicing between animals with low and high IR in spleen and skin (sheet Splicing), and enriched functional clusters under high stringency conditions for those genes using DAVID FAC analysis (sheet GOanalysis). (XLSX)

S8 Table Genes exhibiting alternative promote usage between animals with low and high IR in spleen and skin (sheet PromoterUsage), and enriched functional clusters under high stringency conditions for skin genes using DAVID FAC analysis (sheet GOanalysis). (XLSX)

S9 Table Differential expression results of the genes analysed in spleen and skin samples from animals showing extreme IRs using real-time PCR (P<0.09). (DOCX)
