# Supplementary material for: Transcriptomic Characterization of Innate and Acquired Immune Responses in Red-Legged Partridges (Alectoris rufa): A Resource for Immunoecology and Robustness Selection
Source: PLoS One. 2015 Sep 2;10(9):e0136776. doi: 10.1371/journal.pone.0136776 (PMC4557936; doi:10.1371/journal.pone.0136776)
Supplement: S1 Data — (DOCX) [file pone.0136776.s001.docx]

**Data S1. Non-infectious challenges methodology and timetable**

Because of the requirements to perform challenges with an infectious agent, such as the availability of facilities with special security measures and request official permissions, we performed non-infectious challenges applying two methodologies widely tested in birds that allowed the classification of partridges according to their overall IR against a wide spectrum of pathogens, with the benefit of not representing any health risk to the animals or the farm (Grasman 2010):

*- Sheep red blood cells (SRBC) hemagglutination assay to measure acquire immunity.* This response, which confers long-lasting immunity against pathogens, occurs through the formation of antibodies that recognize a specific and foreign antigen and establish immunological memory. After initial contact with an antigen, the next time that this pathogen infects the organism, memory cells will trigger a specific and faster response against the pathogen. Agglutination tests measure the production of specific antibodies after the injection of foreign antigens, integrating the functions of B lymphocytes, helper T lymphocytes, and macrophages. SRBC from one sheep was preserved in Alsever’s solution (Sigma, Spain). After an initial assay to evaluate the feasibility of the technique procedure described by Grasman (2010), we followed these guidelines except for the intravascular injection of 0.1 ml of a suspension of 1% SRBC; given the difficulties of consistent intravascular inoculation that we found in partridges, we opted for inoculating 0.2 ml of a 25% SRBC suspension into the thigh muscle (Boa-Amponsem *et al.* 2001). Blood plasma was collected at day 0 prior to inoculation of SRCB suspension and 6 days after immunization (Figure), and was serially diluted in duplicate and incubated with SRBC 0.25% for 3 h at 37°C to measure total antibody titers (AbTot). To measure IgG activity, 25 ml of 0.20 M 2-mercaptoethanol (Merck, Germany) in normal saline was added to blood plasma, serially diluted in duplicate and incubated with SRBC 0.25% for 1 h at 37°C. The log_2_ of the reciprocal of the greatest dilution of antibody at which there was a visible complex between antigen and antibody was defined as the titer in both cases. A high titer indicates a strong antibody response to the original immunization.

*- Skin test with phytohemagglutinin (PHA) to measure innate immunity.* Cells and mechanisms of the innate immune system are not specific, recognizing and responding to pathogens in a generic way. This response has no immunological memory and reaches its maximum intensity at 24-48 hours after contact with the infectious agent. The PHA skin response test is an in vivo method that stimulates T lymphocytes to release lymphokines, resulting in an increase in vascular permeability and the influx of a variety of leukocytes. It measures the swelling caused by inflammatory leukocyte and fluid infiltration after an intradermal injection of PHA. A large increase in skin thickness indicates a strong T cell-mediated immune response. In addition to its sensitivity to contaminants, ecological studies have shown that the PHA skin response is positively associated with rates of survival and colonization of new areas in wild birds (Moeller & Cassey 2004; Moeller & Saino, 2004), and lead to birds with an enhanced IR (Sundaresan *et al.* 2005). In this assay, the procedure described by Grasman (2010) was followed except for the concentration of the PHA solution. After an initial trial, we found that the inflammatory response elicited by the 1 mg/ml PHA solution reported by Grasman (2010) was insufficient in partridges to accurately measure differences among individuals. Thus, PHA concentration was increased to 1.6 mg/ml. Phytohemagglutinin-P (Sigma, Spain) was dissolved in sterile phosphate buffer saline (PBS) (AppliChem Panreac Nova Chimica, Spain). Intedigitary skin was measured in duplicate using a pressure-sensitive caliper (Mitutoyo 7313 Dial Thickness Gage, Spain) before injecting 0.1 ml of PHA sub-dermally into one foot and 0.1 ml of PBS into the other foot (day 5) (Figure). Twenty-four hours (±2 h) after injections (day 6), the thickness of each foot interdigitary skin was measured and the mitogen stimulation index was calculated as the increase in interdigitary skin thickness caused by PHA minus the increase caused by PBS.

**Figure**. Timetable of non-infectious challenges with phytohemagglutinin (PHA) and sheep red blood cells (SRBC).

**References**

Boa-Amponsem K, Price SE, Dunnington EA, Siegel PB (2001) Effect of route of inoculation on humoral immune response of White Leghorn chickens selected for high or low antibody response to sheep red blood cells. *Poultry Science*, **80**, 1073-1078.

Grasman KA (2010) In vivo functional tests for assessing immunotoxicity in birds. *Methods in Molecular Biology*, **598**, 387-398.

Moeller AP, Cassey P (2004) On the relationship between T-cell mediated immunity in bird species and the establishment success of introduced populations. *Journal of Animal Ecology*, **73**, 1035-1042.

Moeller AP, Saino N (2004) Immune response and survival. *Oikos*, **104**, 299–304.

Sundaresan NR, Ahmed KA, Saxena VK, *et al.* (2005) Differential expression of inducible nitric oxide synthase and cytokine mRNA in chicken lines divergent for cutaneous hypersensitivity response. *Veterinary Immunology and Immunopathology*, **108**, 373-385.
