# Supplementary figures and images for: Transcriptomic Characterization of Innate and Acquired Immune Responses in Red-Legged Partridges (Alectoris rufa): A Resource for Immunoecology and Robustness Selection
Source: PLoS One. 2015 Sep 2;10(9):e0136776. doi: 10.1371/journal.pone.0136776 (PMC4557936; doi:10.1371/journal.pone.0136776)

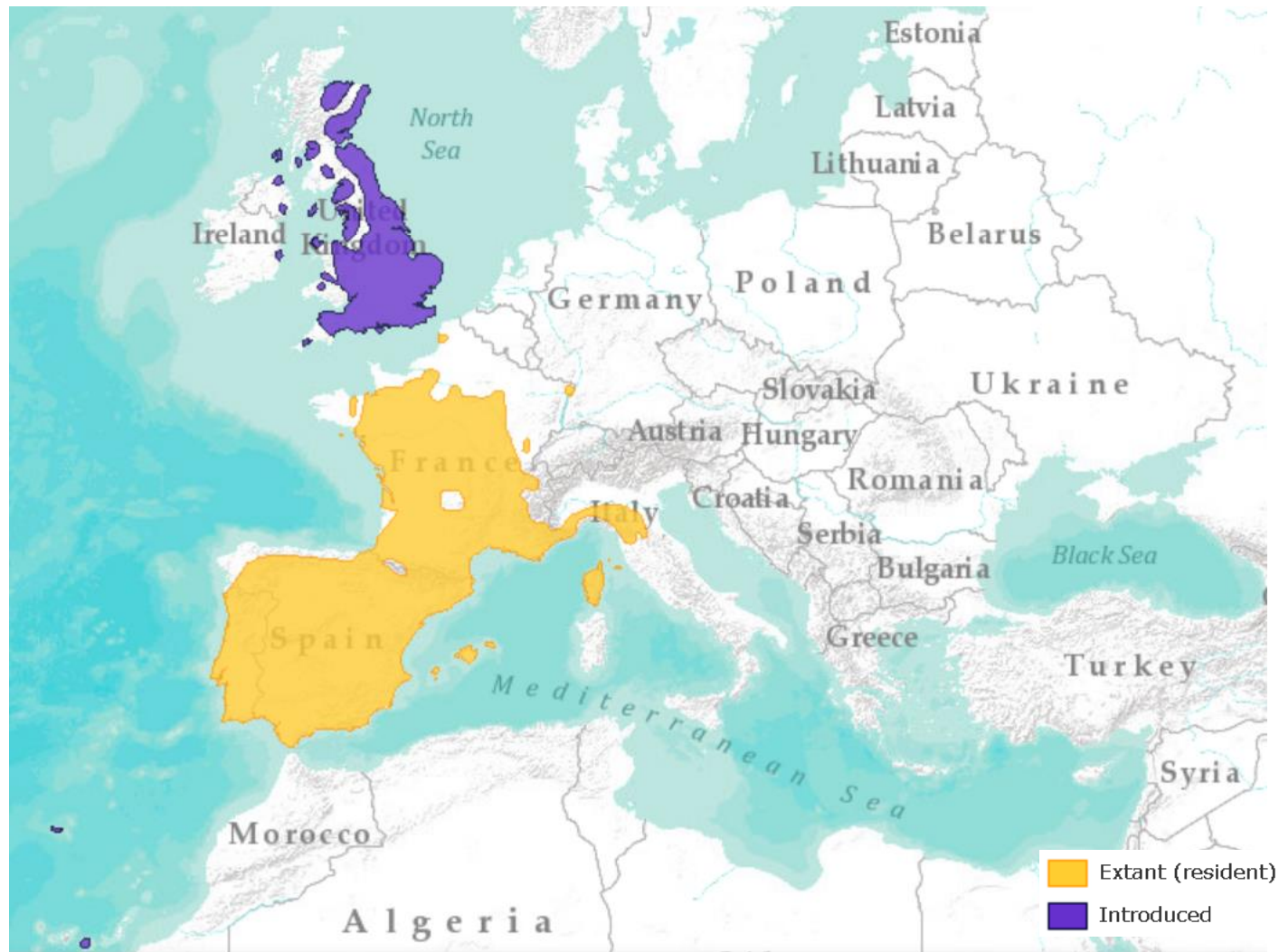

Supplement: S1 Fig — (PDF) [file pone.0136776.s002.pdf]

A1

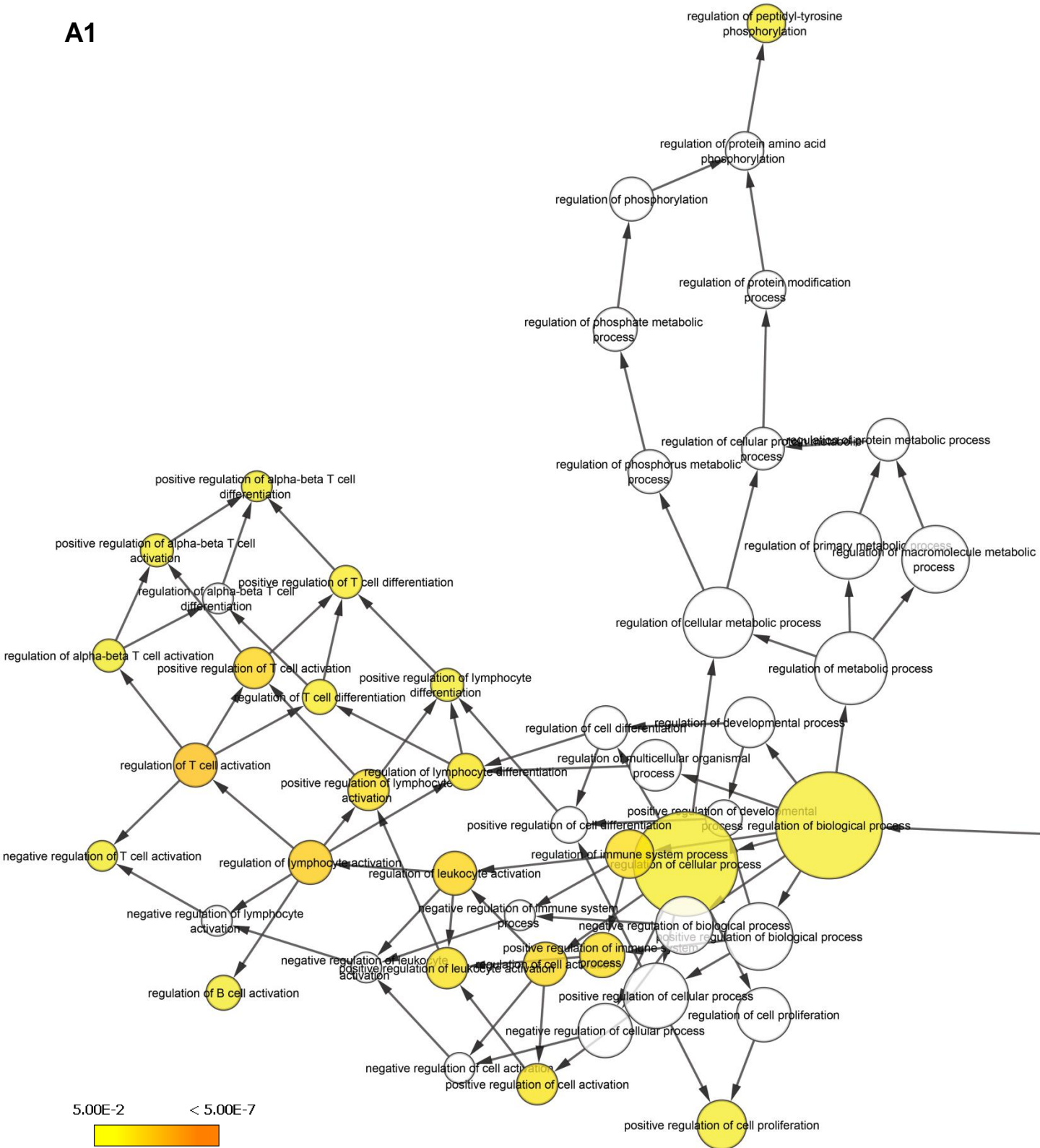

A2

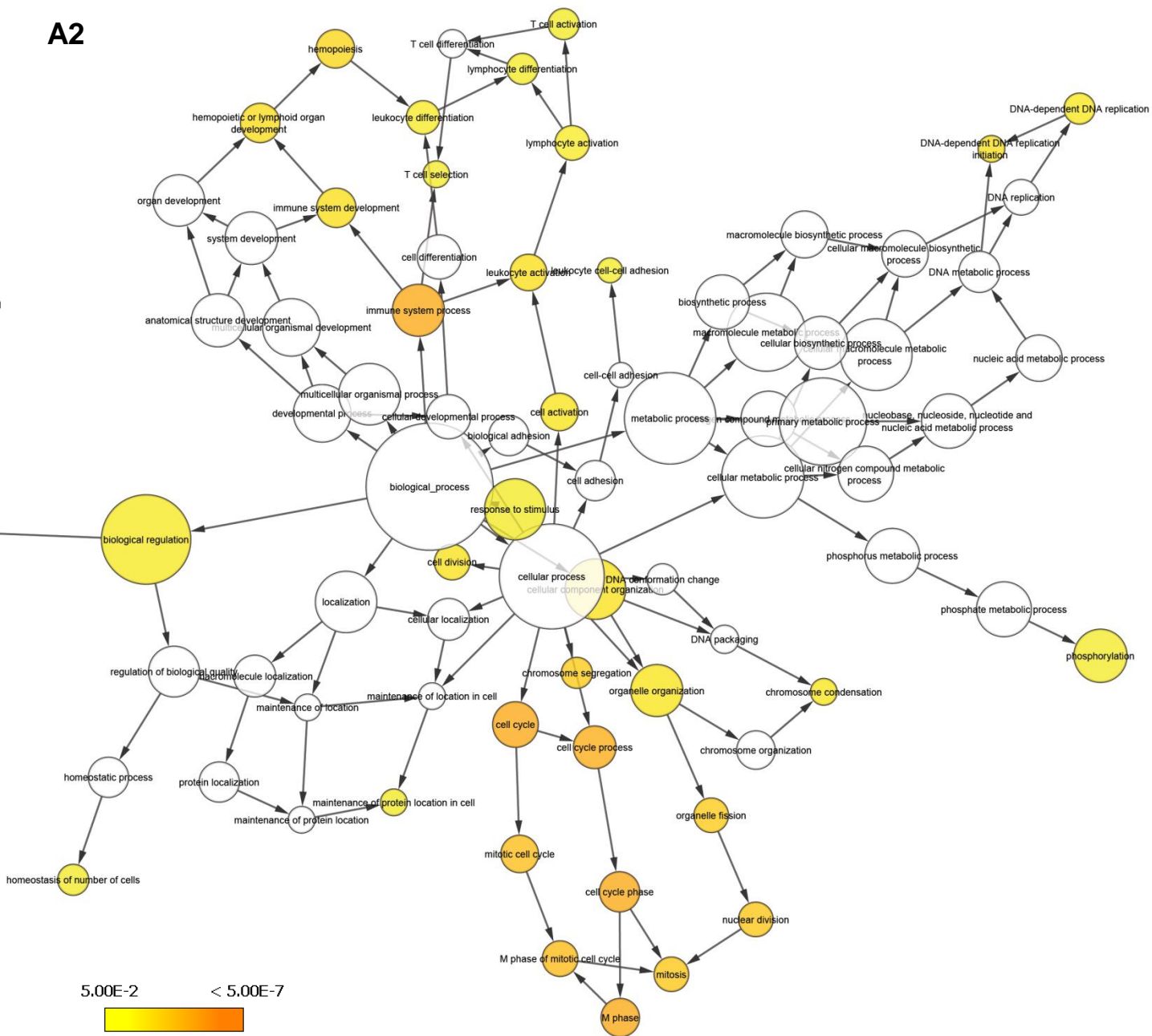

**B**

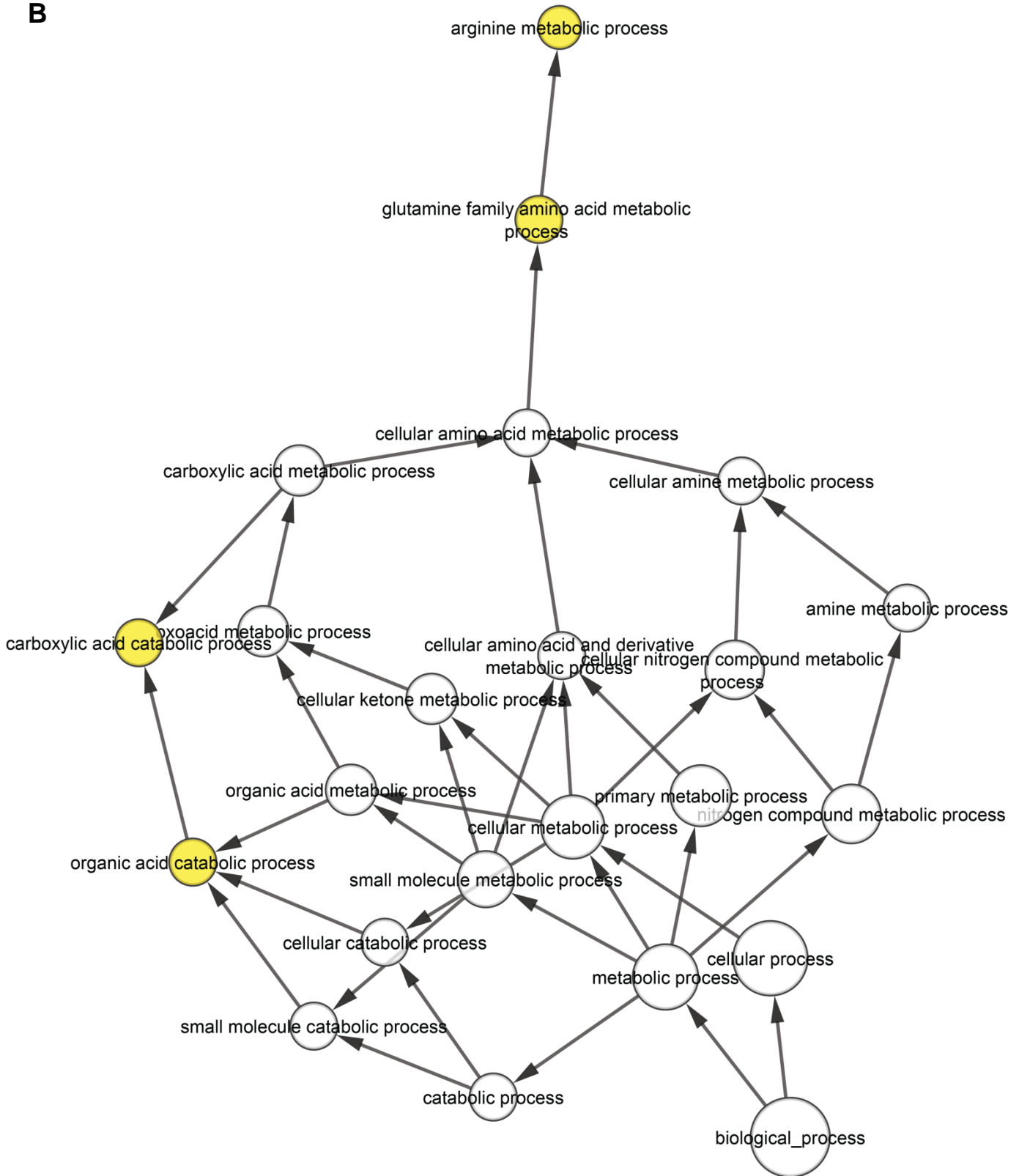

Supplement: S2 Fig — The size of circles is proportional to the number of genes associated with the GO term. The arrows represent the relationship between parent–child terms. The colour scale indicates corrected p-value of enrichment analysis: uncoloured nodes are not overrepresented, but they are the parents of overrepresented categories further down; yellow nodes represent GO categories that are overrepresented at the significance level; for more significant p-values, the node colour gets increasingly more orange. Both A1 and A2 figures are connected by an arrow (*) from ‘biological regulation’ to ‘regulation of biological processes’. (PDF) [file pone.0136776.s003.pdf]
