# Supplementary material for: Transcriptomic Characterization of Innate and Acquired Immune Responses in Red-Legged Partridges (Alectoris rufa): A Resource for Immunoecology and Robustness Selection
Source: PLoS One. 2015 Sep 2;10(9):e0136776. doi: 10.1371/journal.pone.0136776 (PMC4557936; doi:10.1371/journal.pone.0136776)
Supplement: S3 Fig — Red stars indicate the genes differentially expressed included in the pathway. (PDF) [file pone.0136776.s004.pdf]

A

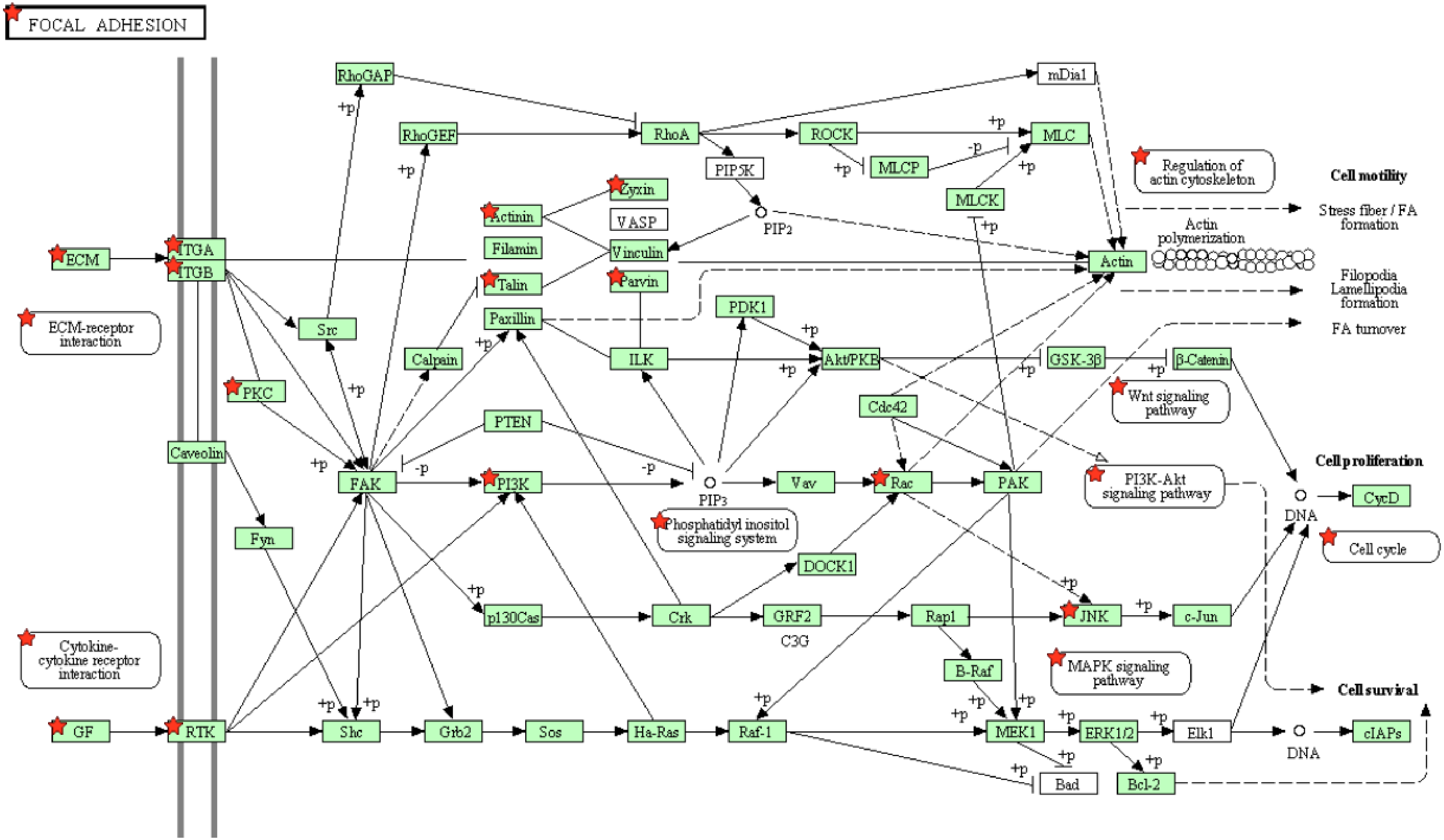
FOCAL ADHESION
ECM
ITGA
ITGB
ECM-receptor interaction
PKC
Caveolin
Fyn
Cytokine-cytokine receptor interaction
GF
RTK
RhoGAP
RhoGEF
Src
FAK
Calpain
Actinin
Zyxin
VASP
Filamin
Vinculin
Talin
Parvin
Paxillin
ILK
PTEN
PI3K
PIP3
Phosphatidyl inositol signaling system
RhoA
PIP5K
PIP2
ROCK
MLCP
MLC
MLCK
mDia1
Actin
Actin polymerization
Regulation of actin cytoskeleton
Cell motility
Stress fiber / FA formation
Filopodia Lamellipodia formation
FA turnover
PDK1
Akt/PKB
GSK-3β
β-Catenin
Wnt signaling pathway
Cdc42
Vav
Rac
PAK
PI3K-Akt signaling pathway
Cell proliferation
DNA
CycD
Cell cycle
DOCK1
p130Cas
Crk
GRF2
C3G
Rap1
JNK
c-Jun
B-Raf
MAPK signaling pathway
Cell survival
Shc
Grb2
Sos
Ha-Ras
Raf-1
MEK1
ERK1/2
Elk1
cIAPs
Bad
Bcl-2
+p
-p

B

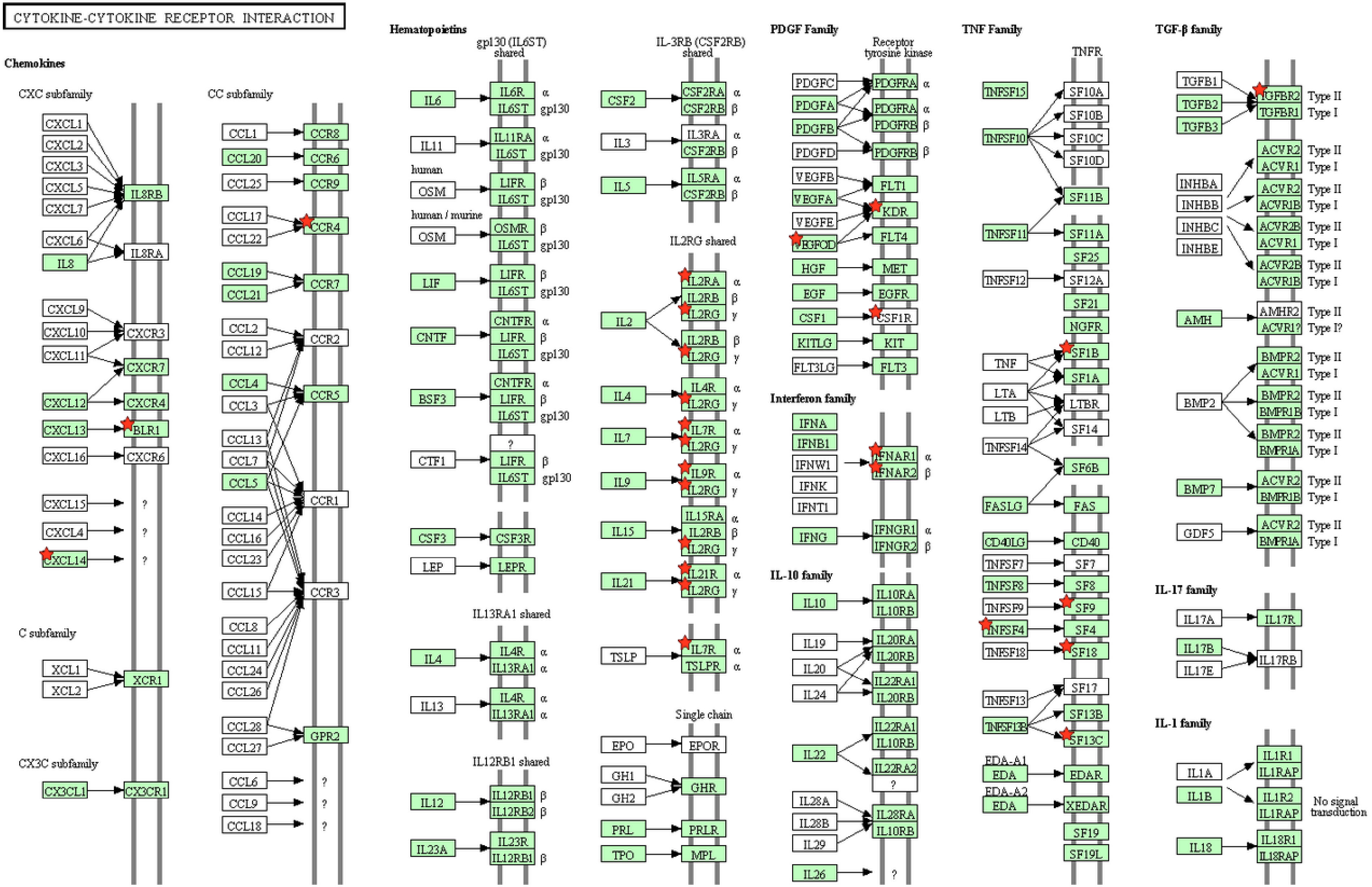
CYTOKINE-CYTOKINE RECEPTOR INTERACTION
Chemokines
CXC subfamily
CXCL1
CXCL2
CXCL3
CXCL5
CXCL7
CXCL6
IL8
IL8RB
IL8RA
CXCL9
CXCL10
CXCL11
CXCR3
CXCR7
CXCL12
CXCR4
CXCL13
BLR1
CXCL16
CXCR6
CXCL15
CXCL4
CXCL14
?
C subfamily
XCL1
XCL2
XCR1
CX3C subfamily
CX3CL1
CX3CR1
CC subfamily
CCL1
CCR8
CCL20
CCR6
CCL25
CCR9
CCL17
CCL22
CCR4
CCL19
CCL21
CCR7
CCL2
CCL12
CCR2
CCL4
CCL3
CCR5
CCL13
CCL7
CCL5
CCL14
CCL16
CCL23
CCR1
CCL15
CCR3
CCL8
CCL11
CCL24
CCL26
CCL28
CCL27
GPR2
CCL6
CCL9
CCL18
Hematopoietins
gp130 (IL6ST) shared
IL6
IL6R
IL6ST
IL11
IL11RA
human
OSM
LIFR
human / murine
OSMR
LIF
CNTF
CNTFR
BSF3
CTF1
α
β
gp130
CSF3
CSF3R
LEP
LEPR
IL13RA1 shared
IL4
IL4R
IL13RA1
IL13
IL12RB1 shared
IL12
IL12RB1
IL12RB2
IL23A
IL23R
IL-3RB (CSF2RB) shared
CSF2
CSF2RA
CSF2RB
IL3
IL3RA
IL5
IL5RA
IL2RG shared
IL2
IL2RA
IL2RB
IL2RG
γ
IL4R
IL7
IL7R
IL9
IL9R
IL15
IL15RA
IL21
IL21R
TSLP
TSLPR
Single chain
EPO
EPOR
GH1
GH2
GHR
PRL
PRLR
TPO
MPL
PDGF Family
Receptor tyrosine kinase
PDGFC
PDGFA
PDGFB
PDGFD
PDGFRA
PDGFRB
VEGFB
VEGFA
VEGFC
VEGFD
FLT1
KDR
FLT4
HGF
MET
EGF
EGFR
CSF1
CSF1R
KITLG
KIT
FLT3LG
FLT3
Interferon family
IFNA
IFNB1
IFNW1
IFNK
IFNT1
IFNAR1
IFNAR2
IFNG
IFNGR1
IFNGR2
IL-10 family
IL10
IL10RA
IL10RB
IL19
IL20
IL24
IL20RA
IL20RB
IL22RA1
IL22
IL22RA2
IL28A
IL28B
IL29
IL28RA
IL26
TNF Family
TNFR
TNFSF15
SF10A
TNFSF10
SF10B
SF10C
SF10D
SF11B
TNFSF11
SF11A
SF25
TNFSF12
SF12A
SF21
NGFR
TNF
SF1B
SF1A
LTA
LTB
LTBR
TNFSF14
SF14
SF6B
FASLG
FAS
CD40LG
CD40
TNFSF7
SF7
TNFSF8
SF8
TNFSF9
SF9
TNFSF4
SF4
TNFSF18
SF18
TNFSF13
SF17
TNFSF13B
SF13B
SF13C
EDA-A1
EDA
EDAR
EDA-A2
XEDAR
SF19
SF19L
TGF-β family
TGFB1
TGFB2
TGFB3
TGFBR2
TGFBR1
Type II
Type I
INHBA
INHBB
INHBC
INHBE
ACVR2
ACVR1
ACVR2B
ACVR1B
AMH
AMHR2
ACVR1?
Type I?
BMP2
BMPR2
BMPR1B
BMPR1A
BMP7
GDF5
IL-17 family
IL17A
IL17R
IL17B
IL17E
IL17RB
IL-1 family
IL1A
IL1B
IL1R1
IL1RAP
IL1R2
No signal transduction
IL18
IL18R1
IL18RAP

C

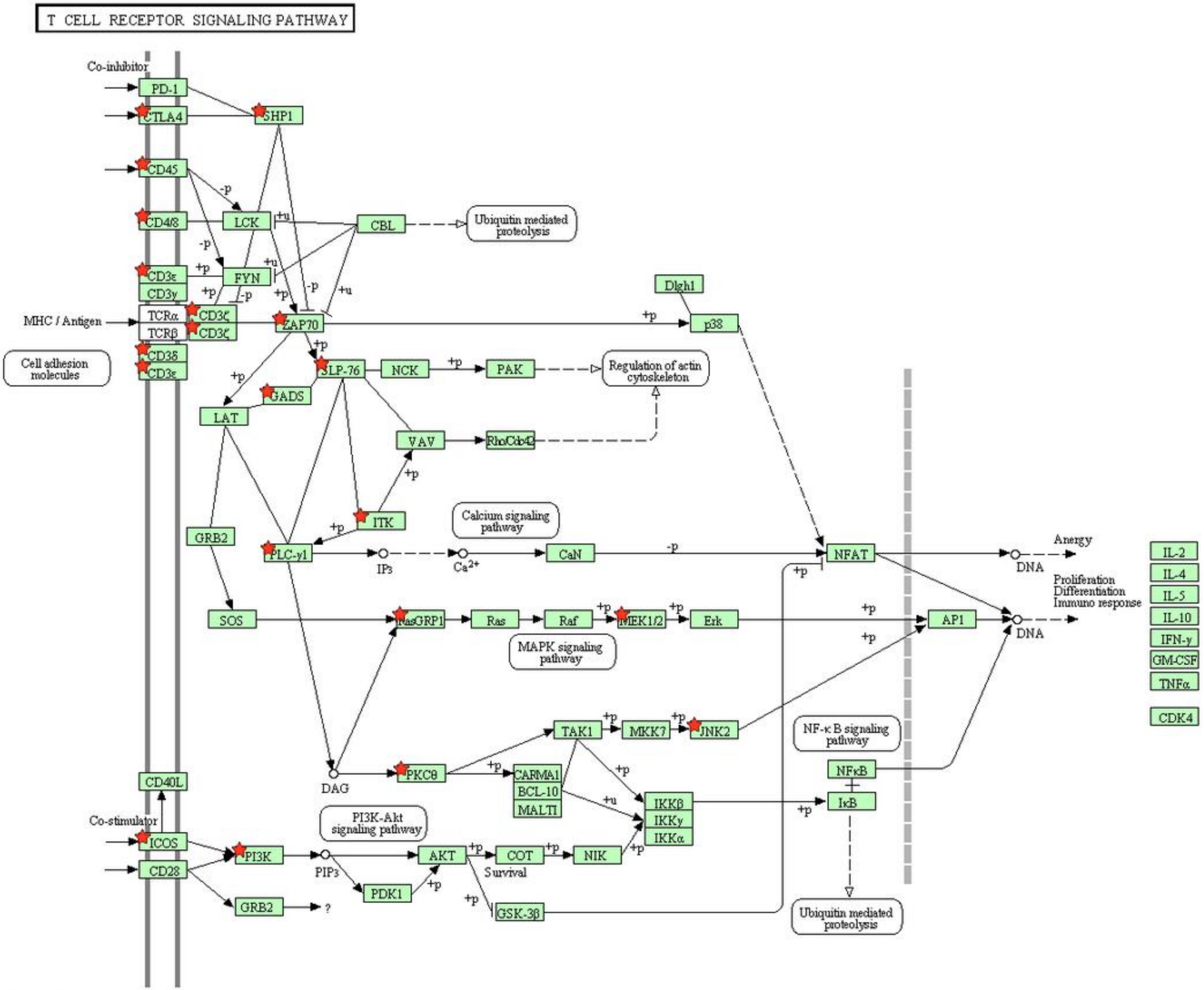

T CELL RECEPTOR SIGNALING PATHWAY
Co-inhibitor
PD-1
CTLA4
SHP1
CD45
CD4/8
LCK
CBL
Ubiquitin mediated proteolysis
CD3ε
CD3γ
FYN
MHC / Antigen
TCRα
TCRβ
CD3ζ
CD3ζ
ZAP70
Dlgh1
p38
CD3δ
CD3ε
Cell adhesion molecules
SLP-76
NCK
PAK
Regulation of actin cytoskeleton
GADS
LAT
VAV
Rho/Cdc42
ITK
Calcium signaling pathway
GRB2
PLC-γ1
IP3
Ca2+
CaN
NFAT
DNA
Anergy
Proliferation
Differentiation
Immuno response
IL-2
IL-4
IL-5
IL-10
IFN-γ
GM-CSF
TNFα
CDK4
SOS
RasGRP1
Ras
Raf
MEK1/2
Erk
AP1
DNA
MAPK signaling pathway
TAK1
MKK7
JNK2
NF-κB signaling pathway
NFκB
IκB
DAG
PKCθ
CARMA1
BCL-10
MALT1
IKKβ
IKKγ
IKKα
CD40L
Co-stimulator
ICOS
CD28
PI3K
PI3K-Akt signaling pathway
PIP3
AKT
COT
NIK
Survival
PDK1
GRB2
GSK-3β
Ubiquitin mediated proteolysis

D

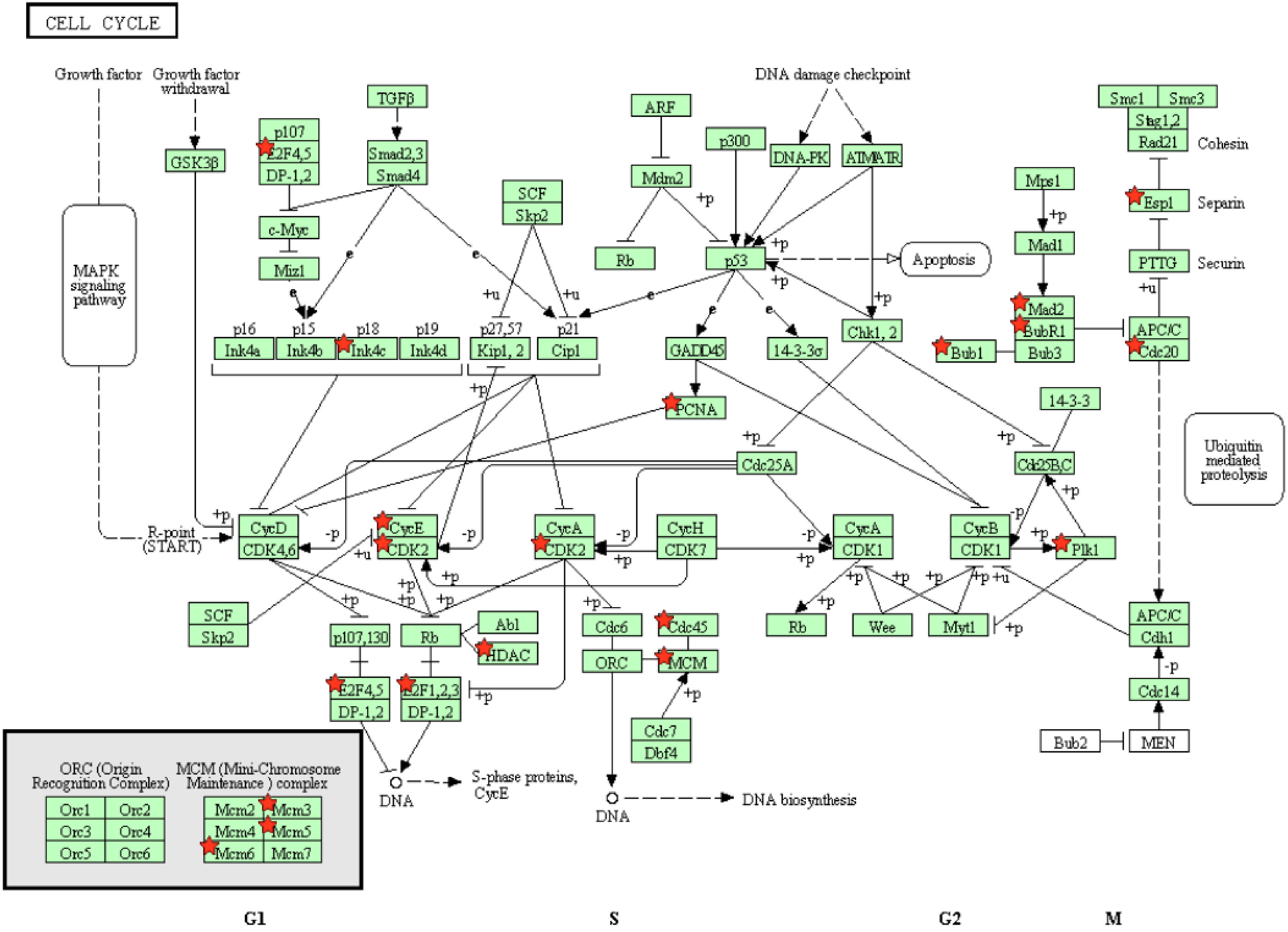
CELL CYCLE
Growth factor
Growth factor withdrawal
GSK3β
MAPK signaling pathway
p107
E2F4,5
DP-1,2
TGFβ
Smad2,3
Smad4
c-Myc
Miz1
SCF
Skp2
ARF
Mdm2
Rb
p300
DNA damage checkpoint
DNA-PK
ATM/ATR
p53
Apoptosis
Mps1
Mad1
Mad2
BubR1
Bub1
Bub3
Smc1
Smc3
Stag1,2
Rad21
Cohesin
Esp1
Separin
PTTG
Securin
APC/C
Cdc20
p16
Ink4a
p15
Ink4b
p18
Ink4c
p19
Ink4d
p27,57
Kip1, 2
p21
Cip1
GADD45
14-3-3σ
Chk1, 2
PCNA
14-3-3
Cdc25A
Cdc25B,C
Ubiquitin mediated proteolysis
R-point (START)
CycD
CDK4,6
CycE
CDK2
CycA
CDK2
CycH
CDK7
CycA
CDK1
CycB
CDK1
Plk1
SCF
Skp2
p107,130
Rb
Abl
HDAC
E2F4,5
DP-1,2
E2F1,2,3
DP-1,2
Cdc6
Cdc45
ORC
MCM
Cdc7
Dbf4
Rb
Wee
Myt1
APC/C
Cdh1
Cdc14
Bub2
MEN
DNA
S-phase proteins, CycE
DNA
DNA biosynthesis
ORC (Origin Recognition Complex)
Orc1
Orc2
Orc3
Orc4
Orc5
Orc6
MCM (Mini-Chromosome Maintenance) complex
Mcm2
Mcm3
Mcm4
Mcm5
Mcm6
Mcm7
G1
S
G2
M

E

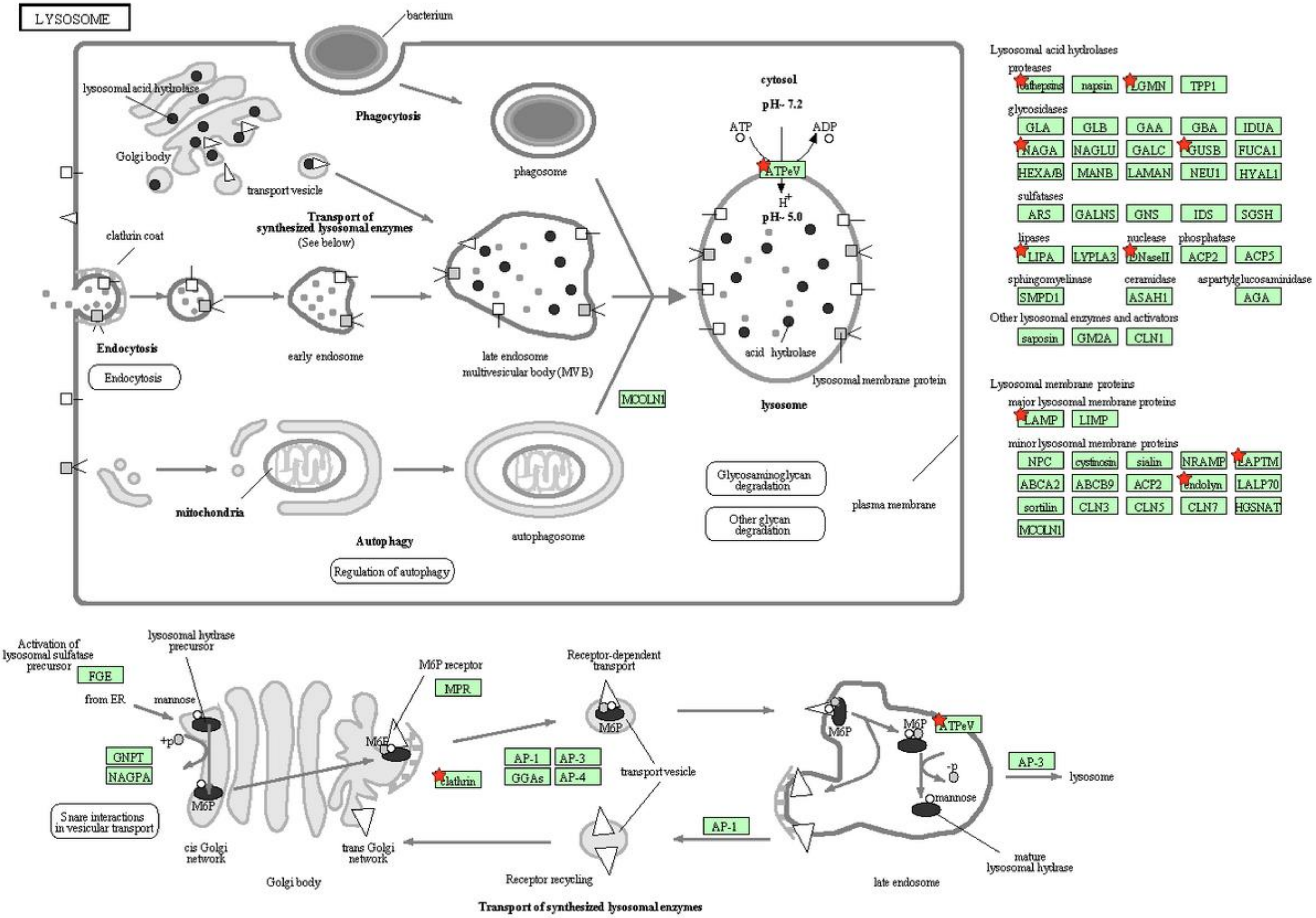

LYSOSOME
bacterium
lysosomal acid hydrolase
Golgi body
Phagocytosis
phagosome
transport vesicle
Transport of
synthesized lysosomal enzymes
(See below)
clathrin coat
Endocytosis
Endocytosis
early endosome
late endosome
multivesicular body (MVB)
cytosol
pH~ 7.2
ATP
ADP
ATPeV
H+
pH~ 5.0
acid hydrolase
lysosomal membrane protein
lysosome
MCOLN1
mitochondria
Autophagy
Regulation of autophagy
autophagosome
Glycosaminoglycan degradation
Other glycan degradation
plasma membrane
Lysosomal acid hydrolases
proteases
cathepsins
napsin
LGMN
TPP1
glycosidases
GLA
GLB
GAA
GBA
IDUA
NAGA
NAGLU
GALC
GUSB
FUCA1
HEXA/B
MANB
LAMAN
NEU1
HYAL1
sulfatases
ARS
GALNS
GNS
IDS
SGSH
lipases
LIPA
LYPLA3
nuclease
DNaseII
phosphatase
ACP2
ACP5
sphingomyelinase
SMPD1
ceramidase
ASAH1
aspartylglucosaminidase
AGA
Other lysosomal enzymes and activators
saposin
GM2A
CLN1
Lysosomal membrane proteins
major lysosomal membrane proteins
LAMP
LIMP
minor lysosomal membrane proteins
NPC
cystinosin
sialin
NRAMP
LAPTM
ABCA2
ABCB9
ACP2
endolyn
LALP70
sortilin
CLN3
CLN5
CLN7
HGSNAT
MCOLN1
Activation of lysosomal sulfatase precursor
FGE
lysosomal hydrase precursor
from ER
mannose
+p
GNPT
NAGPA
M6P
Snare interactions in vesicular transport
cis Golgi network
Golgi body
M6P receptor
MPR
M6P
clathrin
trans Golgi network
Receptor-dependent transport
M6P
AP-1
AP-3
GGAs
AP-4
transport vesicle
Receptor recycling
AP-1
M6P
M6P
ATPeV
-p
mannose
AP-3
lysosome
mature lysosomal hydrase
late endosome
Transport of synthesized lysosomal enzymes

F

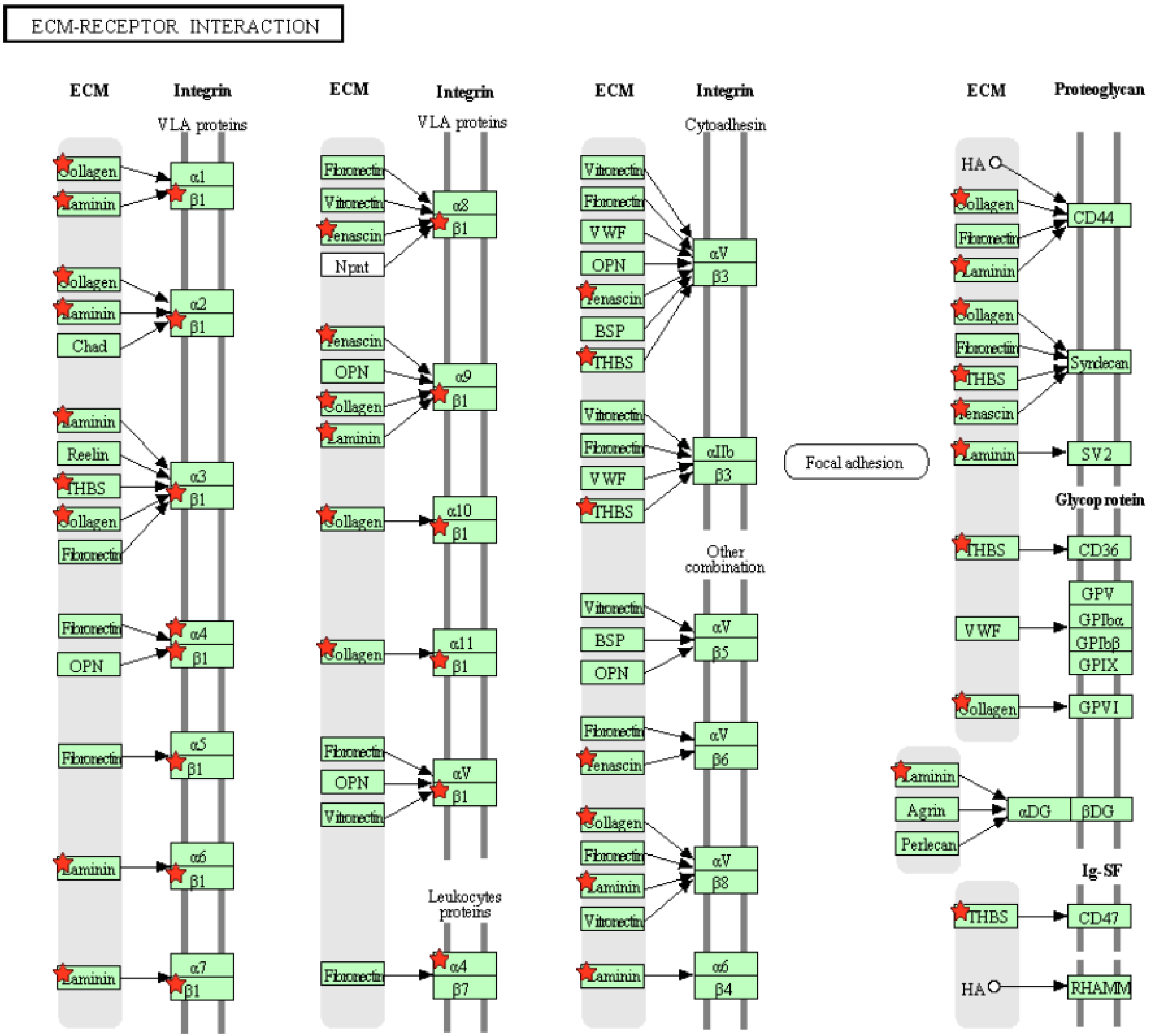
ECM-RECEPTOR INTERACTION
ECM
Integrin
VLA proteins
Collagen
Laminin
Chad
Reelin
THBS
Fibronectin
OPN
α1
α2
α3
α4
α5
α6
α7
β1
Vitronectin
Tenascin
Npnt
α8
α9
α10
α11
αV
Leukocytes proteins
β7
Cytoadhesin
VWF
BSP
β3
αIIb
Other combination
β5
β6
β8
β4
Focal adhesion
Proteoglycan
HA
CD44
Syndecan
SV2
Glycoprotein
CD36
GPV
GPIbα
GPIbβ
GPIX
GPVI
Agrin
Perlecan
αDG
βDG
Ig-SF
CD47
RHAMM

G

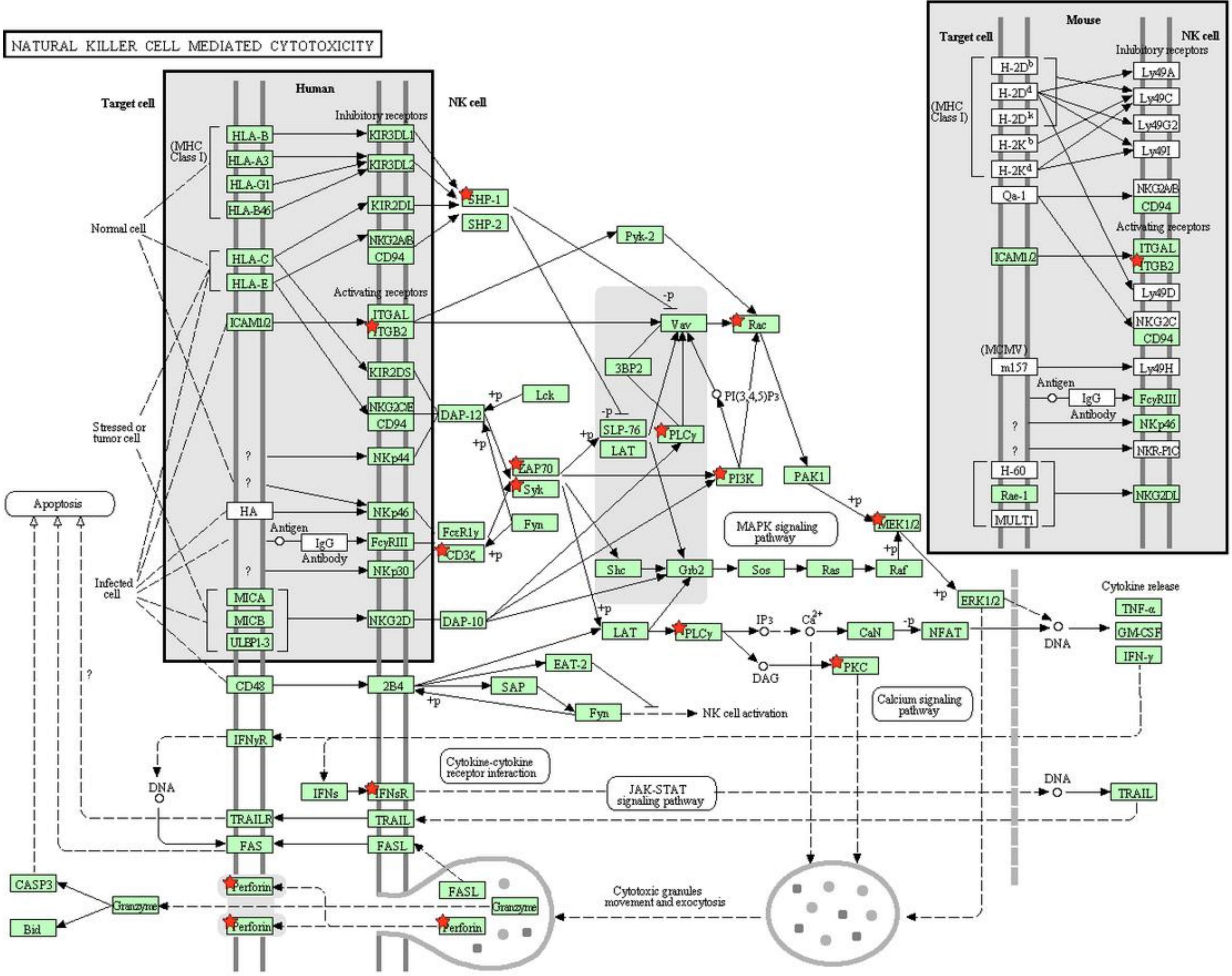
NATURAL KILLER CELL MEDIATED CYTOTOXICITY
Human
Target cell
NK cell
Inhibitory receptors
(MHC Class I)
HLA-B
HLA-A3
HLA-G1
HLA-B46
HLA-C
HLA-E
KIR3DL1
KIR3DL2
KIR2DL
NKG2A/B
CD94
SHP-1
SHP-2
Normal cell
Activating receptors
ICAM1/2
ITGAL
ITGB2
KIR2DS
NKG2C/E
CD94
NKp44
NKp46
NKp30
HA
Antigen
IgG
Antibody
FcγRIII
MICA
MICB
ULBP1-3
NKG2D
Stressed or tumor cell
Infected cell
Apoptosis
DAP-12
DAP-10
FcεR1γ
CD3ζ
Lck
ZAP70
Syk
Fyn
Pyk-2
Vav
Rac
3BP2
SLP-76
LAT
PLCγ
PI(3,4,5)P3
PI3K
PAK1
MEK1/2
MAPK signaling pathway
Shc
Grb2
Sos
Ras
Raf
ERK1/2
IP3
Ca2+
CaN
NFAT
DAG
PKC
DNA
Cytokine release
TNF-α
GM-CSF
IFN-γ
CD48
2B4
EAT-2
SAP
Fyn
NK cell activation
Calcium signaling pathway
IFNγR
IFNs
IFNαR
Cytokine-cytokine receptor interaction
JAK-STAT signaling pathway
TRAIL
TRAILR
FAS
FASL
Perforin
Granzyme
CASP3
Bid
Cytotoxic granules movement and exocytosis
Mouse
Target cell
NK cell
Inhibitory receptors
(MHC Class I)
H-2D^b
H-2D^d
H-2D^k
H-2K^b
H-2K^d
Qa-1
Ly49A
Ly49C
Ly49G2
Ly49I
NKG2A/B
CD94
Activating receptors
ICAM1/2
ITGAL
ITGB2
Ly49D
NKG2C
CD94
(MCMV)
m157
Ly49H
Antigen
IgG
Antibody
FcγRIII
NKp46
NKR-P1C
H-60
Rae-1
MULT1
NKG2DL

H

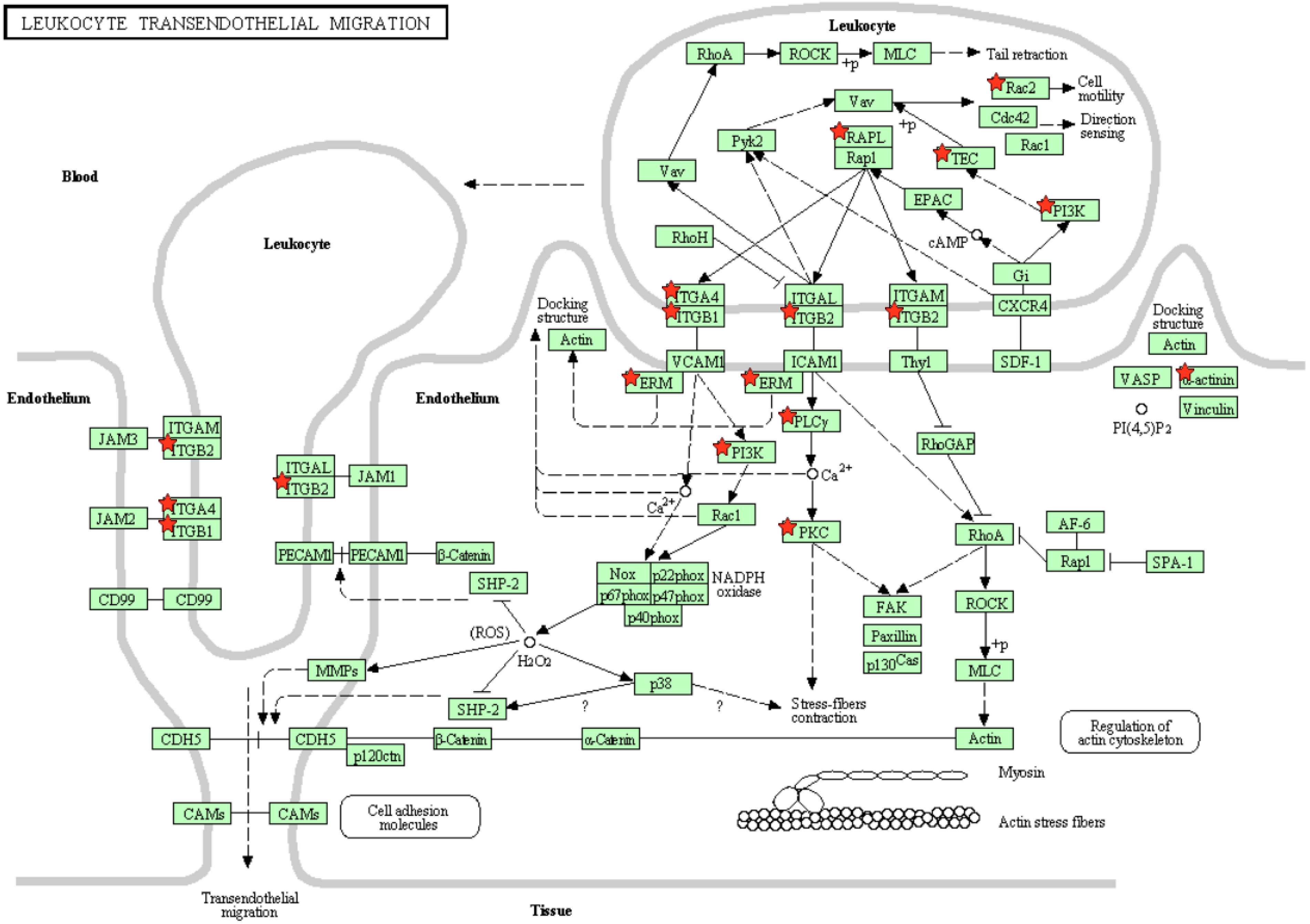
LEUKOCYTE TRANSENDOTHELIAL MIGRATION
Leukocyte
RhoA
ROCK
+p
MLC
Tail retraction
Rac2
Cell motility
Vav
Cdc42
Direction sensing
Rac1
Pyk2
RAPL
Rap1
TEC
EPAC
PI3K
cAMP
Gi
CXCR4
SDF-1
RhoH
ITGA4
ITGB1
ITGAL
ITGB2
ITGAM
Thy1
VCAM1
ICAM1
ERM
PLCγ
PI3K
Ca2+
Rac1
PKC
RhoGAP
RhoA
AF-6
Rap1
SPA-1
ROCK
MLC
Actin
FAK
Paxillin
p130Cas
Docking structure
Actin
VASP
α-actinin
Vinculin
PI(4,5)P2
Blood
Leukocyte
Endothelium
JAM3
JAM2
CD99
JAM1
PECAM1
β-Catenin
SHP-2
Nox
p22phox
p67phox
p47phox
p40phox
NADPH oxidase
(ROS)
H2O2
MMPs
p38
SHP-2
Stress-fibers contraction
CDH5
p120ctn
α-Catenin
CAMs
Cell adhesion molecules
Regulation of actin cytoskeleton
Myosin
Actin stress fibers
Transendothelial migration
Tissue

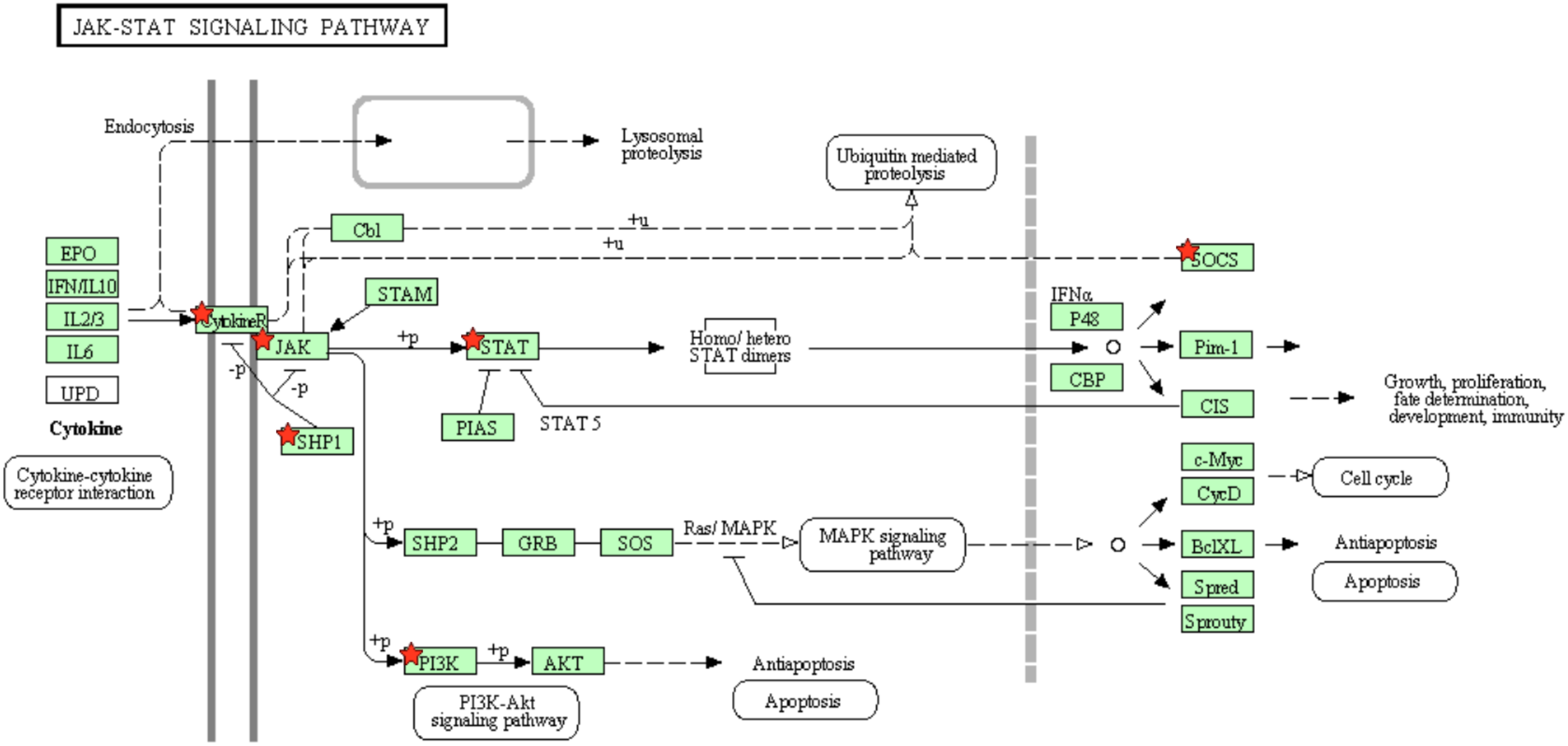
JAK-STAT SIGNALING PATHWAY
Endocytosis
Lysosomal proteolysis
Ubiquitin mediated proteolysis
Cbl
+u
+u
SOCS
EPO
IFN/IL10
IL2/3
IL6
UPD
Cytokine
CytokineR
STAM
JAK
+p
-p
-p
STAT
Homo/ hetero STAT dimers
IFNα
P48
CBP
Pim-1
CIS
Growth, proliferation, fate determination, development, immunity
SHP1
PIAS
STAT 5
Cytokine-cytokine receptor interaction
c-Myc
CycD
Cell cycle
+p
SHP2
GRB
SOS
Ras/ MAPK
MAPK signaling pathway
BclXL
Antiapoptosis
Apoptosis
Spred
Sprouty
+p
PI3K
+p
AKT
Antiapoptosis
Apoptosis
PI3K-Akt signaling pathway

J

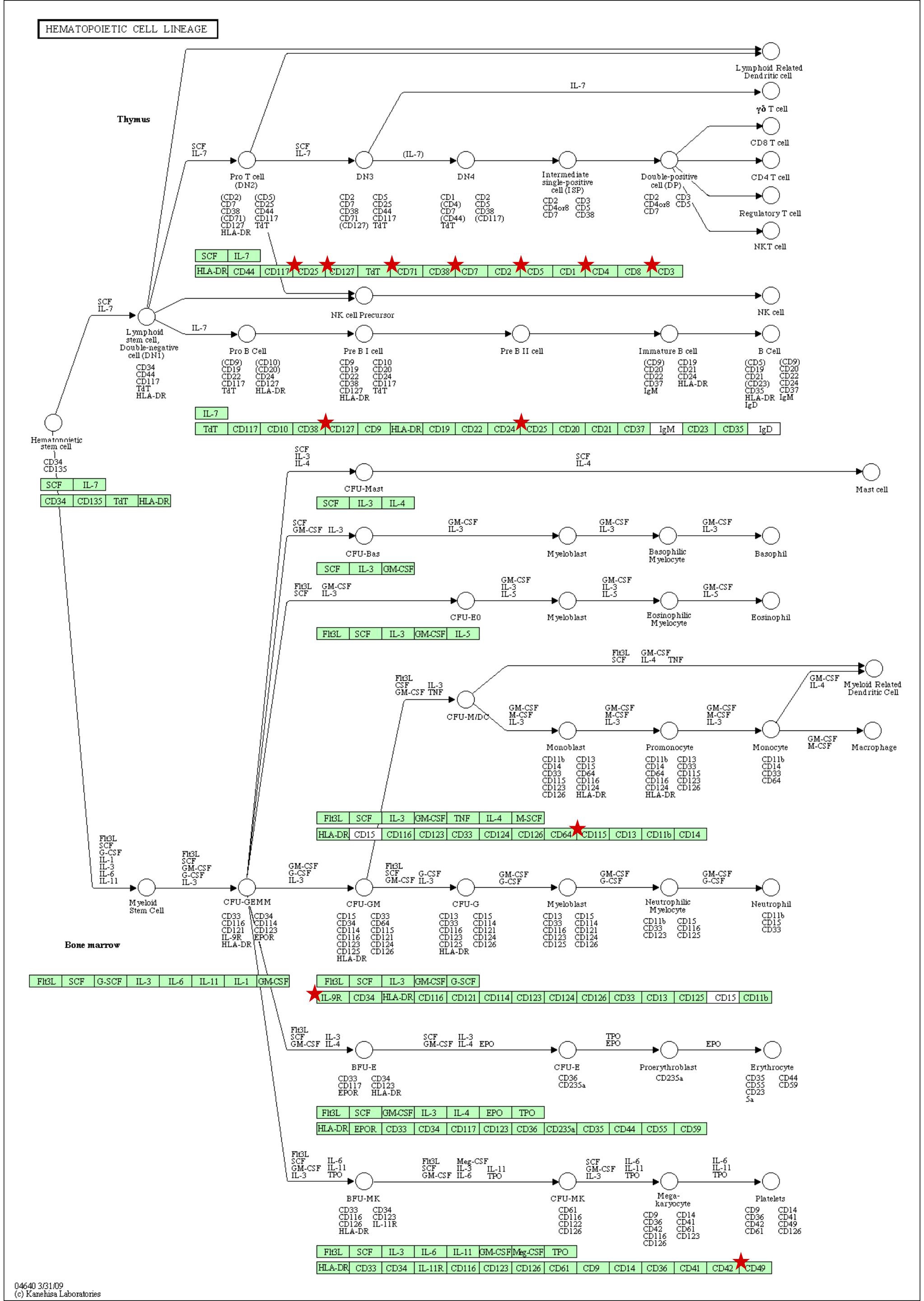

HEMATOPOIETIC CELL LINEAGE
Thymus
Lymphoid Related Dendritic cell
IL-7
γδ T cell
CD8 T cell
CD4 T cell
Regulatory T cell
NKT cell
SCF IL-7
Pro T cell (DN2)
(CD2) CD7 CD38 (CD71) CD127 HLA-DR
(CD5) CD25 CD44 CD117 TdT
DN3
CD2 CD7 CD38 CD71 (CD127)
CD5 CD25 CD44 CD117 TdT
(IL-7)
DN4
CD1 (CD4) CD7 (CD44) TdT
CD2 CD5 CD38 (CD117)
Intermediate single-positive cell (ISP)
CD2 CD4or8 CD7
CD3 CD5 CD38
Double-positive cell (DP)
CD2 CD4or8 CD7
CD3 CD5
SCF | IL-7
HLA-DR | CD44 | CD117 | CD25 | CD127 | TdT | CD71 | CD38 | CD7 | CD2 | CD5 | CD1 | CD4 | CD8 | CD3
NK cell Precursor
NK cell
Lymphoid stem cell, Double-negative cell (DN1)
CD34 CD44 CD117 TdT HLA-DR
Pro B Cell
(CD9) CD19 CD22 CD117 TdT
(CD10) (CD20) CD24 CD127 HLA-DR
Pre B I cell
CD9 CD19 CD22 CD38 CD127 HLA-DR
CD10 CD20 CD24 CD117 TdT
Pre B II cell
Immature B cell
(CD9) CD20 CD22 CD37 IgM
CD19 CD21 CD24 HLA-DR
B Cell
(CD5) CD19 CD21 (CD23) CD35 HLA-DR IgD
(CD9) CD20 CD22 CD24 CD37 IgM
IL-7
TdT | CD117 | CD10 | CD38 | CD127 | CD9 | HLA-DR | CD19 | CD22 | CD24 | CD25 | CD20 | CD21 | CD37 | IgM | CD23 | CD35 | IgD
Hematopoietic stem cell
CD34 CD135
SCF | IL-7
CD34 | CD135 | TdT | HLA-DR
SCF IL-3 IL-4
CFU-Mast
SCF IL-4
Mast cell
SCF | IL-3 | IL-4
SCF GM-CSF IL-3
CFU-Bas
GM-CSF IL-3
Myeloblast
Basophilic Myelocyte
Basophil
SCF | IL-3 | GM-CSF
Flt3L SCF GM-CSF IL-3
CFU-E0
GM-CSF IL-3 IL-5
Eosinophilic Myelocyte
GM-CSF IL-5
Eosinophil
Flt3L | SCF | IL-3 | GM-CSF | IL-5
Flt3L SCF GM-CSF IL-4 TNF
Flt3L CSF GM-CSF IL-3 TNF
CFU-M/DC
Myeloid Related Dendritic Cell
GM-CSF M-CSF IL-3
Monoblast
CD11b CD14 CD33 CD115 CD123 CD126
CD13 CD15 CD64 CD116 CD124 HLA-DR
Promonocyte
CD11b CD14 CD64 CD116 CD124 HLA-DR
CD13 CD33 CD115 CD123 CD126
Monocyte
CD11b CD14 CD33 CD64
GM-CSF M-CSF
Macrophage
Flt3L | SCF | IL-3 | GM-CSF | TNF | IL-4 | M-SCF
HLA-DR | CD15 | CD116 | CD123 | CD33 | CD124 | CD126 | CD64 | CD115 | CD13 | CD11b | CD14
Flt3L SCF G-CSF IL-1 IL-3 IL-6 IL-11
Myeloid Stem Cell
Flt3L SCF GM-CSF G-CSF IL-3
CFU-GEMM
CD33 CD116 CD121 IL-9R HLA-DR
CD34 CD114 CD123 EPOR
GM-CSF G-CSF IL-3
CFU-GM
CD15 CD34 CD114 CD116 CD123 CD125 HLA-DR
CD33 CD64 CD115 CD121 CD124 CD126
Flt3L SCF GM-CSF G-CSF IL-3
CFU-G
CD13 CD33 CD116 CD123 CD125 HLA-DR
CD15 CD114 CD121 CD124 CD126
GM-CSF G-CSF
Myeloblast
CD13 CD33 CD116 CD123 CD125
CD15 CD114 CD121 CD124 CD126
Neutrophilic Myelocyte
CD11b CD33 CD123
CD15 CD116 CD125
Neutrophil
CD11b CD15 CD33
Bone marrow
Flt3L | SCF | G-SCF | IL-3 | IL-6 | IL-11 | IL-1 | GM-CSF
Flt3L | SCF | IL-3 | GM-CSF | G-SCF
IL-9R | CD34 | HLA-DR | CD116 | CD121 | CD114 | CD123 | CD124 | CD126 | CD33 | CD13 | CD125 | CD15 | CD11b
Flt3L SCF GM-CSF IL-3 IL-4
BFU-E
CD33 CD117 EPOR
CD34 CD123 HLA-DR
SCF GM-CSF IL-3 IL-4 EPO
CFU-E
CD36 CD235a
TPO EPO
Proerythroblast
CD235a
EPO
Erythrocyte
CD35 CD55 CD235a
CD44 CD59
Flt3L | SCF | GM-CSF | IL-3 | IL-4 | EPO | TPO
HLA-DR | EPOR | CD33 | CD34 | CD117 | CD123 | CD36 | CD235a | CD35 | CD44 | CD55 | CD59
Flt3L SCF GM-CSF IL-3 IL-6 IL-11 TPO
BFU-MK
CD33 CD116 CD126 HLA-DR
CD34 CD123 IL-11R
Flt3L SCF GM-CSF Meg-CSF IL-3 IL-6 IL-11 TPO
CFU-MK
CD61 CD116 CD122 CD126
SCF GM-CSF IL-3 IL-6 IL-11 TPO
Mega-karyocyte
CD9 CD36 CD42 CD116 CD126
CD14 CD41 CD61 CD123
IL-6 IL-11 TPO
Platelets
CD9 CD36 CD42 CD61
CD14 CD41 CD49 CD126
Flt3L | SCF | IL-3 | IL-6 | IL-11 | GM-CSF | Meg-CSF | TPO
HLA-DR | CD33 | CD34 | IL-11R | CD116 | CD123 | CD126 | CD61 | CD9 | CD14 | CD36 | CD41 | CD42 | CD49
04640 3/31/09
(c) Kanehisa Laboratories

K

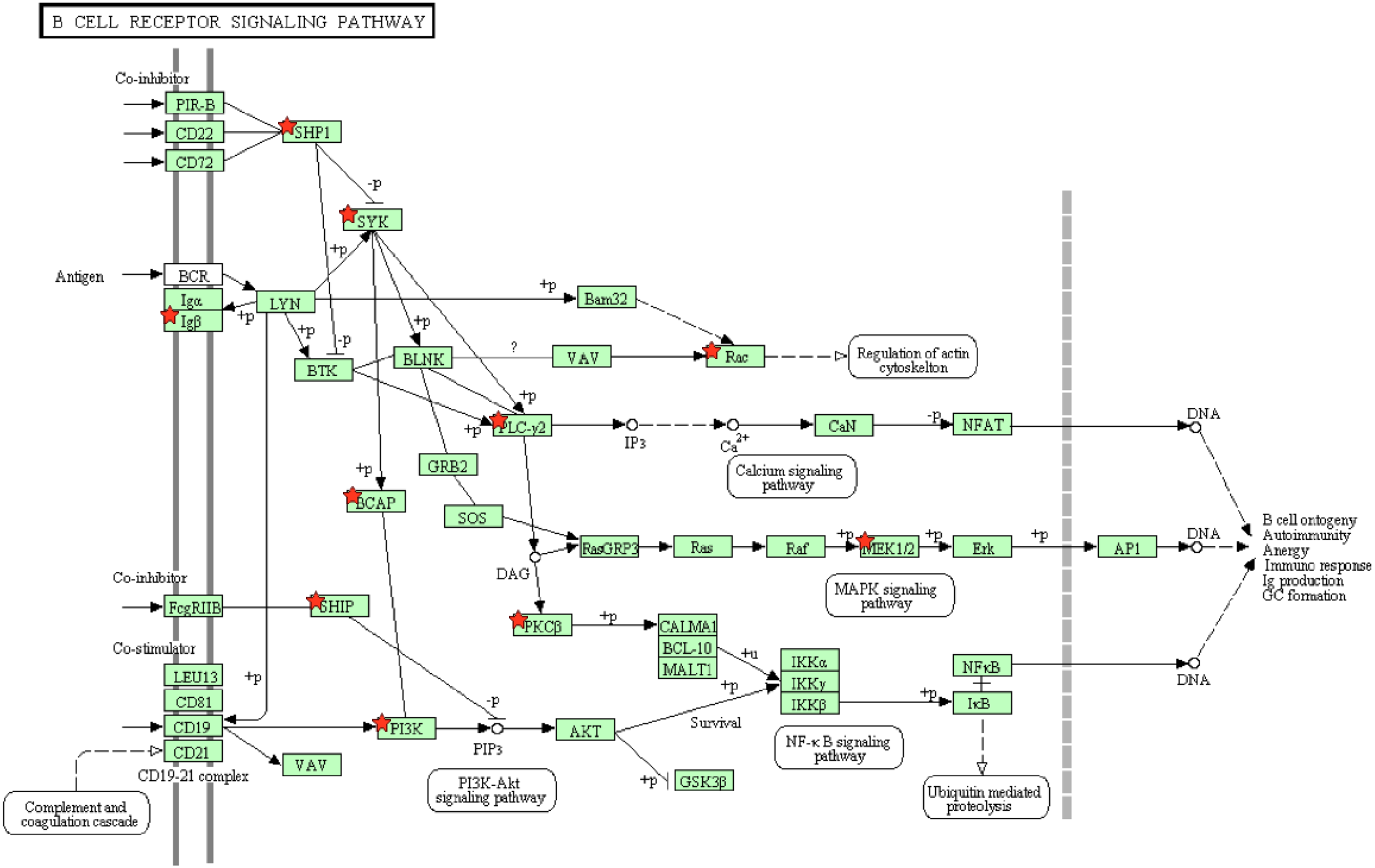
B CELL RECEPTOR SIGNALING PATHWAY
Co-inhibitor
PIR-B
CD22
CD72
SHP1
-p
SYK
+p
Antigen
BCR
Igα
Igβ
LYN
+p
Bam32
+p
-p
BTK
BLNK
?
VAV
Rac
Regulation of actin cytoskelton
PLC-γ2
IP3
Ca2+
CaN
NFAT
DNA
Calcium signaling pathway
GRB2
BCAP
SOS
RasGRP3
Ras
Raf
MEK1/2
Erk
AP1
DAG
MAPK signaling pathway
B cell ontogeny
Autoimmunity
Anergy
Immuno response
Ig production
GC formation
Co-inhibitor
FcgRIIB
SHIP
PKCβ
CALMA1
BCL-10
MALT1
+u
IKKα
IKKγ
IKKβ
NFκB
IκB
Co-stimulator
LEU13
CD81
CD19
CD21
CD19-21 complex
PI3K
PIP3
AKT
Survival
VAV
GSK3β
PI3K-Akt signaling pathway
NF-κB signaling pathway
Ubiquitin mediated proteolysis
Complement and coagulation cascade

L

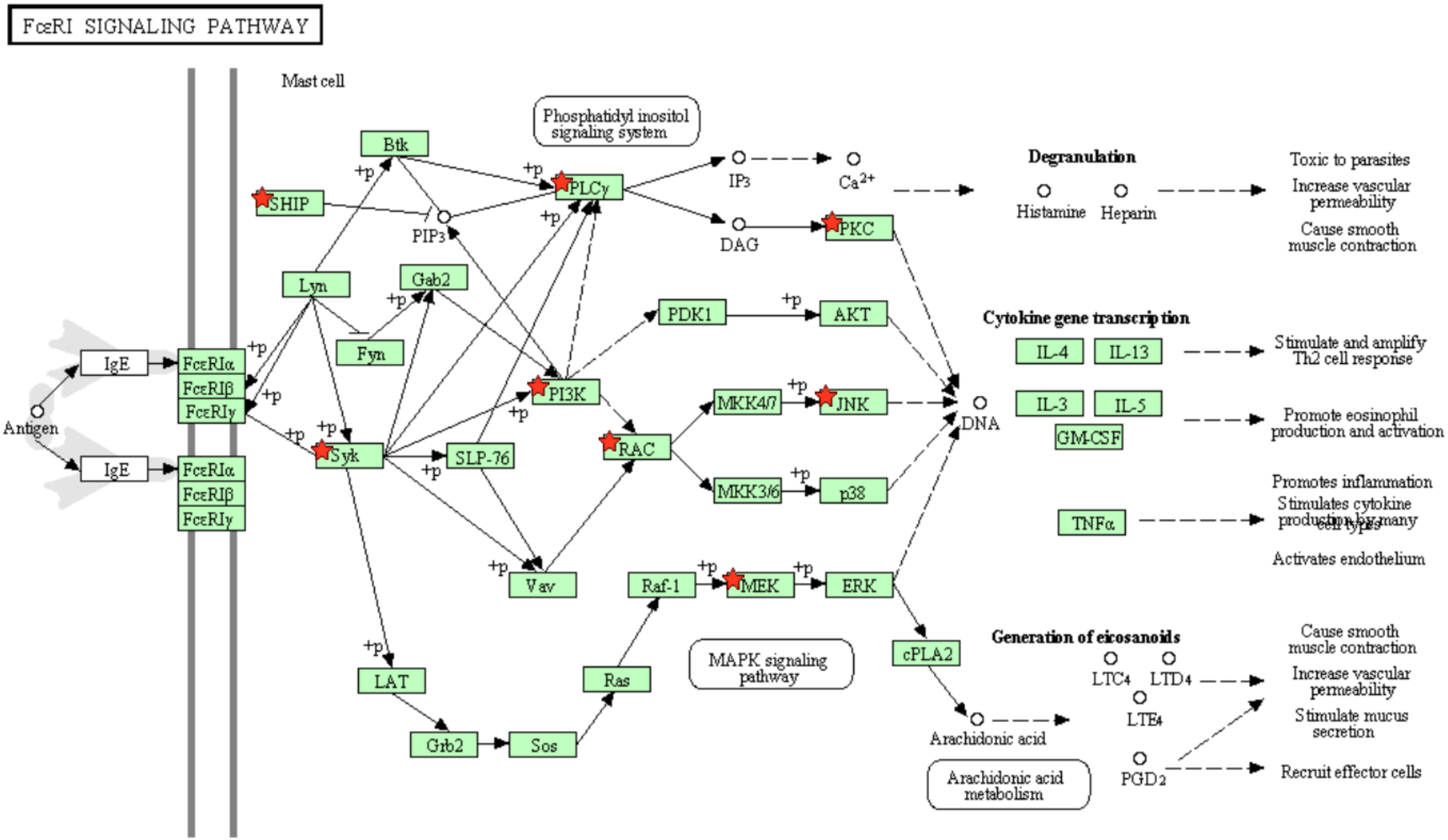
FcεRI SIGNALING PATHWAY
Mast cell
Phosphatidyl inositol signaling system
Btk
SHIP
PLCγ
IP3
Ca2+
DAG
PKC
PIP3
Degranulation
Histamine
Heparin
Toxic to parasites
Increase vascular permeability
Cause smooth muscle contraction
Lyn
Gab2
Fyn
PDK1
AKT
Cytokine gene transcription
IL-4
IL-13
Stimulate and amplify Th2 cell response
IgE
FcεRIα
FcεRIβ
FcεRIγ
Antigen
PI3K
MKK4/7
JNK
DNA
IL-3
IL-5
GM-CSF
Promote eosinophil production and activation
Syk
SLP-76
RAC
MKK3/6
p38
Promotes inflammation
TNFα
Activates endothelium
Vav
Raf-1
MEK
ERK
Generation of eicosanoids
cPLA2
LTC4
LTD4
LTE4
PGD2
Cause smooth muscle contraction
Increase vascular permeability
Stimulate mucus secretion
Recruit effector cells
MAPK signaling pathway
LAT
Ras
Grb2
Sos
Arachidonic acid
Arachidonic acid metabolism
+p

M

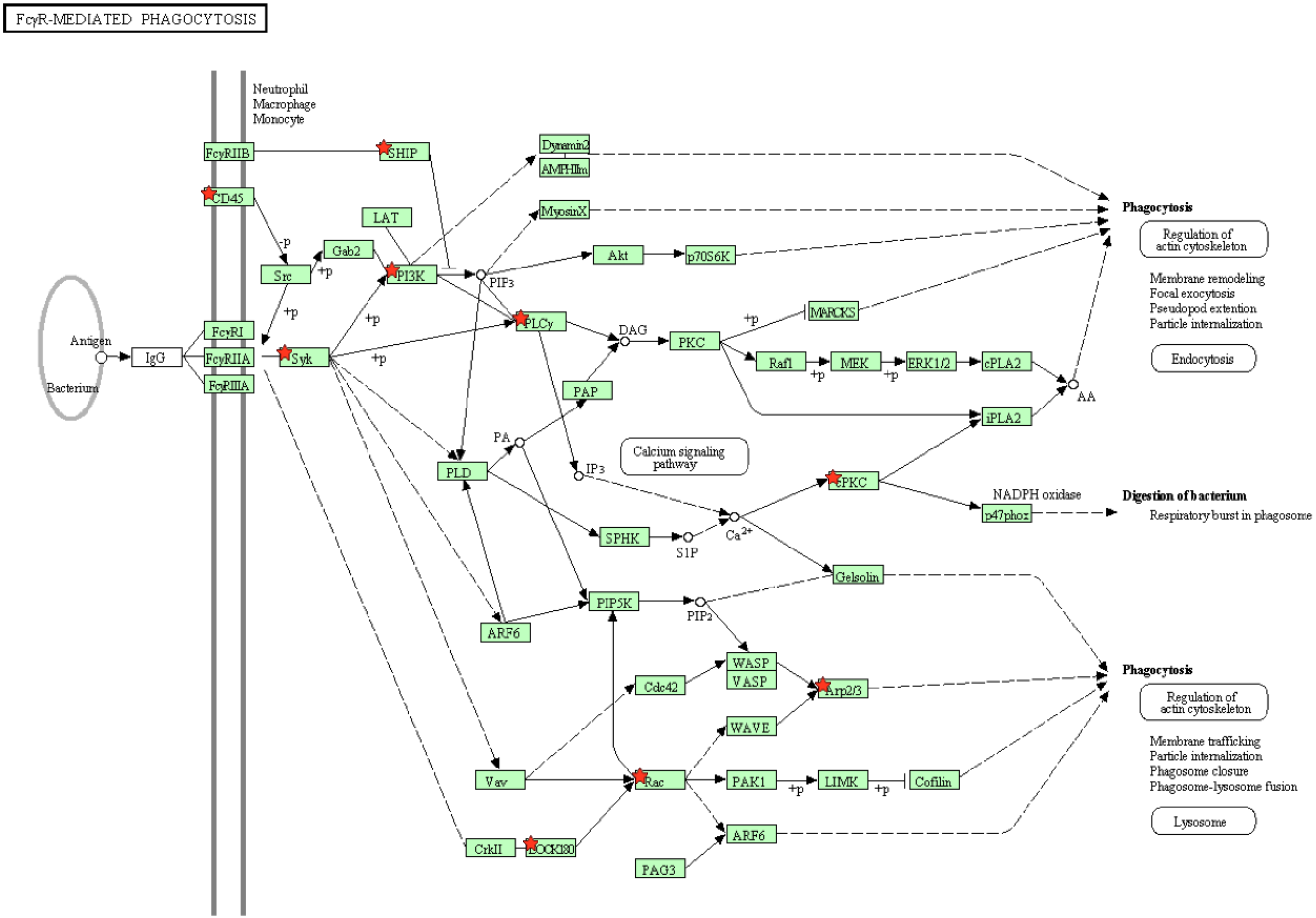
FcγR-MEDIATED PHAGOCYTOSIS
Neutrophil
Macrophage
Monocyte
Antigen
Bacterium
IgG
FcγRIIB
CD45
FcγRI
FcγRIIA
FcγRIIIA
SHIP
LAT
Gab2
Src
PI3K
Syk
PIP3
Dynamin2
AMPHIIm
MyosinX
Akt
p70S6K
PLCγ
DAG
PKC
MARCKS
Raf1
MEK
ERK1/2
cPLA2
iPLA2
AA
PAP
PA
PLD
IP3
Calcium signaling pathway
cPKC
NADPH oxidase
p47phox
SPHK
S1P
Ca2+
Gelsolin
PIP5K
PIP2
ARF6
WASP
VASP
Cdc42
Arp2/3
WAVE
Vav
Rac
PAK1
LIMK
Cofilin
CrkII
DOCK180
ARF6
PAG3
Phagocytosis
Regulation of actin cytoskeleton
Membrane remodeling
Focal exocytosis
Pseudopod extention
Particle internalization
Endocytosis
Digestion of bacterium
Respiratory burst in phagosome
Phagocytosis
Regulation of actin cytoskeleton
Membrane trafficking
Particle internalization
Phagosome closure
Phagosome-lysosome fusion
Lysosome

N

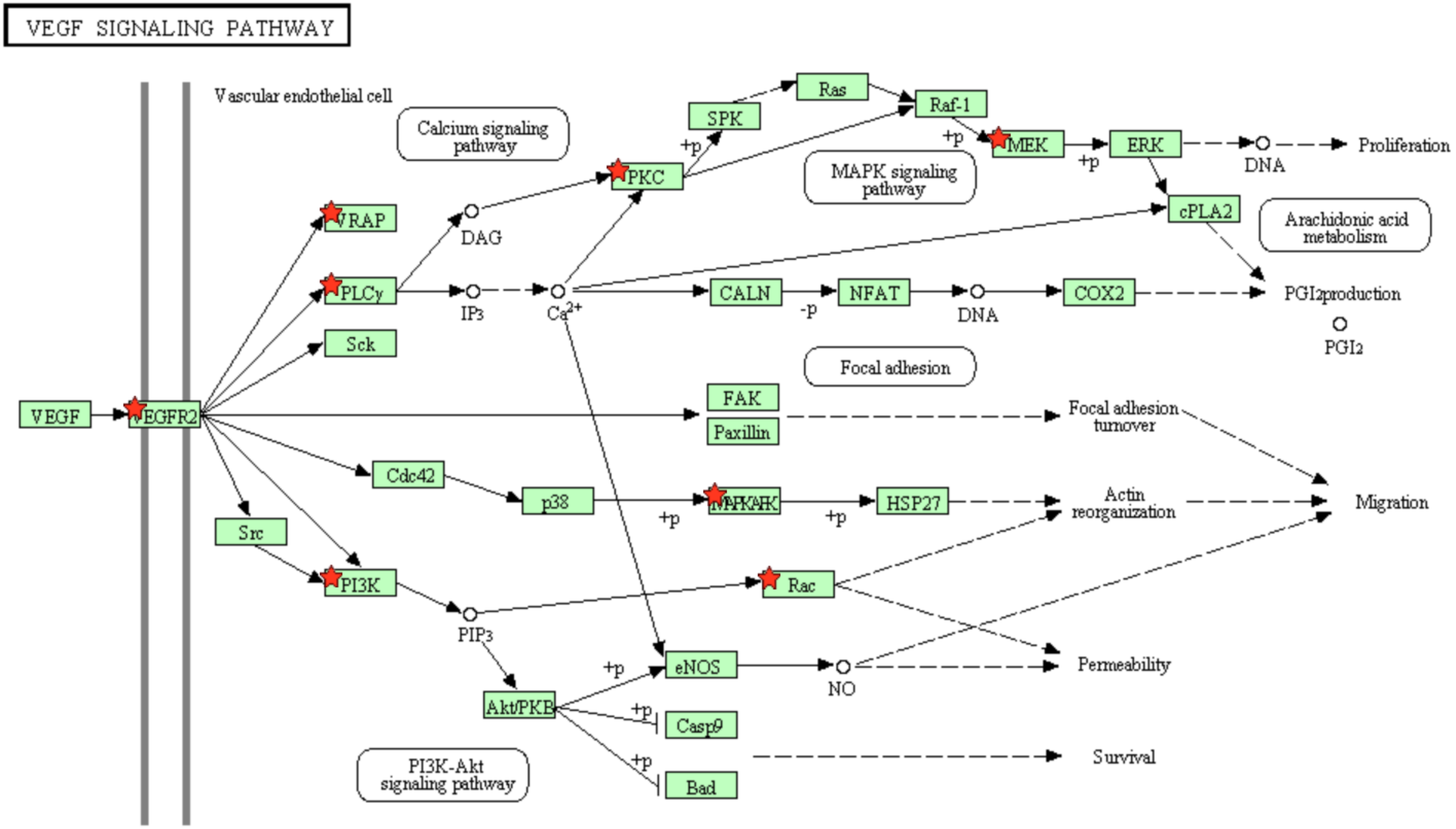

VEGF SIGNALING PATHWAY
Vascular endothelial cell
Calcium signaling pathway
MAPK signaling pathway
Arachidonic acid metabolism
Focal adhesion
PI3K-Akt signaling pathway
VEGF
VEGFR2
VRAP
PLCγ
Sck
Src
Cdc42
p38
MAPKAPK
HSP27
PI3K
PIP3
Akt/PKB
eNOS
Casp9
Bad
Rac
FAK
Paxillin
DAG
IP3
Ca2+
PKC
SPK
Ras
Raf-1
MEK
ERK
DNA
Proliferation
cPLA2
CALN
NFAT
COX2
PGI2production
PGI2
NO
Focal adhesion turnover
Actin reorganization
Migration
Permeability
Survival
+p
-p
