# Supplementary material for: Transcriptomic Characterization of Innate and Acquired Immune Responses in Red-Legged Partridges (Alectoris rufa): A Resource for Immunoecology and Robustness Selection
Source: PLoS One. 2015 Sep 2;10(9):e0136776. doi: 10.1371/journal.pone.0136776 (PMC4557936; doi:10.1371/journal.pone.0136776)
Supplement: S1 Table — (DOCX) [file pone.0136776.s005.docx]

**Table S1** Housekeeping and target genes used in the real-time PCR assay indicating locus symbol, GenBank accession number, and primer sequence.

|  | **Locus Symbol** | **GenBank AccessionN^o^** | **Forward primer (5' 🡪 3')** | **Reverse primer (5' 🡪 3')** |
| --- | --- | --- | --- | --- |
| House-keeping | *ACTB* | L08165 | CACAGATCATGTTTGAGACCTT | CATCACAATACCAGTGGTACG |
|  | *GAPDH* | K01458 | GGGAAGCTTACTGGAATGGCT | GGCAGGTCAGGTCAACAACA |
|  | *G6PDH* | AI981686 | CGGGAACCAAATGCACTTCGT | GGCTGCCGTAGAGGTATGGGA |
| Target genes | *ADAMTSL1* |  | GGTGTCTGAGAAAGTTGAGCA | AGGCAACACTCTTAGCTCCA |
|  | *ATP12A* |  | GCCACAATGATGCCAATG | CATCGGTGTGCTCTTCTTCA |
|  | *AVD* |  | GACCATCGGGACTGTGAAC | GCTGTTACGGCTGTGAGGTA |
|  | *CD3E* |  | GCTCCATTTGCTGCCATAC | GCTGCGAAGGAAGAAAGAAC |
|  | *CD7* |  | ACAGTTGTCCCCAGGAGTGT | AAATGACCTCTCCCAAGCAG |
|  | *CTSD* |  | GTCCTGGGCTGAAGCAGTA | GCCCCTCAACTCCAAAACT |
|  | *GSAP* |  | GCAGTTTTGAGTAGCCACCAT | GTGTTGGAAACCCAGGTCTGT |
|  | *MAD2L1* |  | CCATGCCTAATCCCAAGACT | GTTCAAGCCTTTTCCTGCTC |
|  | *NOV* |  | CCCCACACAACACCAAAAC | TCTAACGGCTGGAAGAAAGC |
|  | *SOX13* |  | CGAAGGATGAGAGGAGGAAG | CTTGCTGATGCTGGAGTTGT |
|  | *SPTSSA* |  | ACCATCACCAACGGAACA | GCTGACATAGGCCCACATAA |
|  | *UBASH3A* |  | CGAGGTGAAGATAGCTTGTGG | CAGCGAACTGCTTTCTGTGT |
