# Supplementary material for: Transcriptomic Characterization of Innate and Acquired Immune Responses in Red-Legged Partridges (Alectoris rufa): A Resource for Immunoecology and Robustness Selection
Source: PLoS One. 2015 Sep 2;10(9):e0136776. doi: 10.1371/journal.pone.0136776 (PMC4557936; doi:10.1371/journal.pone.0136776)
Supplement: S9 Table — (DOCX) [file pone.0136776.s013.docx]

**Table S9** Differential expression results of the genes analysed in spleen and skin samples from animals showing extreme IRs using real-time PCR (*P<0.09*).

| **Tissue** | **Gene** | **Type** | **Expression** | **Std. Error** | **95% C.I.** | **P(H1)** | **Result** |
| --- | --- | --- | --- | --- | --- | --- | --- |
| Spleen | *ACTB* | REF | 1.115 |  |  |  |  |
|  | *GAPDH* | REF | 1.058 |  |  |  |  |
|  | *G6PDH* | REF | 0.848 |  |  |  |  |
|  | *AVD* | TRG | 1.41 | 0.844 - 2.745 | 0.486 - 4.134 | 0.08 | UP |
|  | *CTSD* | TRG | 0.638 | 0.368 - 0.986 | 0.248 - 1.305 | 0.615 |  |
|  | *NOV* | TRG | 1.372 | 0.810 - 2.550 | 0.544 - 3.364 | 0.089 | UP |
|  | *SOX13* | TRG | -1.88 | 0.199 - 1.409 | 0.088 - 5.122 | 0.091 | DOWN |
|  | *SPTSSA* | TRG | -2.00 | 0.179 - 1.151 | 0.106 - 1.492 | 0.005 | DOWN |
| Skin | *ACTB* | REF | 1.17 |  |  |  |  |
|  | *GAPDH* | REF | 0.86 |  |  |  |  |
|  | *G6PDH* | REF | 0.994 |  |  |  |  |
|  | *ADAMTSL1* | TRG | -2.488 | 0.163 - 0.842 | 0.107 - 2.146 | 0.004 | DOWN |
|  | *ATP12A* | TRG | -8.000 | 0.022 - 0.793 | 0.009 - 6.456 | 0.002 | DOWN |
|  | *AVD* | TRG | 1.623 | 0.788 - 4.299 | 0.610 - 5.648 | 0.080 | UP |
|  | *CD3E* | TRG | 3.855 | 1.088 - 12.810 | 0.253 - 19.892 | 0.002 | UP |
|  | *CD7* | TRG | 3.807 | 1.045 - 14.018 | 0.155 - 24.242 | 0.006 | UP |
|  | *CTSD* | TRG | 0.531 | 0.185 - 1.505 | 0.099 - 4.083 | 0.06 | UP |
|  | *GSAP* | TRG | 1.568 | 0.906 - 2.910 | 0.633 - 5.103 | 0.041 | UP |
|  | *MAD2L1* | TRG | 1.948 | 0.955 - 4.004 | 0.352 - 7.510 | 0.033 | UP |
|  | *UBASH3A* | TRG | 4.79 | 1.621 - 14.418 | 0.391 - 19.862 | 0 | UP |
